# Association Between Daily Internet Use and Intrinsic Capacity Among Middle-Aged and Older Adults in China: Large Prospective Cohort Study

**DOI:** 10.2196/54200

**Published:** 2024-11-12

**Authors:** Xing-Ling Chen, Jin Li, Shu-Ning Sun, Qiang-Qiang Zhao, Sheng-Rong Lin, Ling-Jun Wang, Zhong-Qi Yang, Shi-Hao Ni, Lu Lu

**Affiliations:** 1 State Key Laboratory of Traditional Chinese Medicine Syndrome The First Affiliated Hospital Guangzhou University of Chinese Medicine Guangzhou China; 2 Lingnan Medical Research Center Guangzhou University of Chinese Medicine Guangzhou China; 3 University Key Laboratory of Traditional Chinese Medicine Prevention and Treatment of Chronic Heart Failure Guangdong Province Guangzhou China; 4 Guangzhou Key Laboratory for Chinese Medicine Prevention and Treatment of Chronic Heart Failure Guangzhou University of Chinese Medicine Guangzhou China

**Keywords:** daily internet use, intrinsic capacity, IC, middle-aged and older adult, healthy aging, social participation

## Abstract

**Background:**

Intrinsic capacity (IC), as a comprehensive measure of an individual’s functional ability, has gained prominence in the framework for healthy aging introduced by the World Health Organization (WHO). As internet usage continues to integrate into daily life, it is imperative to scrutinize the association between internet use and IC to effectively promote healthy aging among the middle-aged and older population.

**Objective:**

This study aimed to investigate whether daily internet use in middle-aged and older adults delays or accelerates the decline in IC.

**Methods:**

Participants included in the China Health and Retirement Longitudinal Study (CHARLS) comprised individuals aged ≥45 years residing in China. We analyzed 4 years of CHARLS data from the first wave (May 2011-March 2012) to the third wave (July 2015-January 2016). Data from the first and third waves were used for longitudinal studies. Self-reported data encompassed internet use, frequency of use, and demographic baseline characteristics. In addition, the IC evaluation involved physical examination and blood test data. Initially, linear regression was used to assess the relationship between daily internet use and IC, followed by regression splines to explore potential nonlinear associations. Subgroup and sensitivity analyses were used to investigate the heterogeneity of IC in specific conditions and the robustness of our results. Mediation effect analysis was conducted to identify the factors that mediate the relationship between daily internet use and IC, focusing on social participation, physical activity, and health status.

**Results:**

Among the 12,826 participants included in the longitudinal analyses, 12,305 (95.9%) did not use the internet, while 521 (4.1%) reported daily internet use with a mean age of 52.62 (SD 7.67) years. After adjusting for demographic variables, socioeconomic factors, lifestyle behaviors, and health conditions and examining the impact of daily internet use and frequency on changes in IC, our findings indicated important associations. Specifically, daily internet use is significantly linked to a slower decline in IC over time (marginal effect 1.58, 95% CI 1.03-2.12; *P*<.001). Individuals with moderate and regular internet use frequency exhibit higher levels of maintenance in IC (marginal effect 0.74, 95% CI 0.45-1.03, *P*<.001). In addition, the relationship between IC changes and internet use frequency demonstrated a nonlinear inverted U-shaped curve (nonlinear *P*=.003). Subgroup analysis further revealed that improvements in IC vary based on age and gender. Furthermore, mediation analysis denoted that more than 28.78% (95% CI 21.24-40.33) of the observed association is mediated by social participation (*P*<.001).

**Conclusions:**

The findings of our research underscore the potential benefits of consistent and moderate internet use in promoting and preserving IC, particularly in cognitive capacity, sensory, vitality, and locomotion. The observed effects may be related to social participation. These insights offer valuable guidance for crafting strategies aimed at fostering healthy aging within the middle-aged and older adult demographics.

## Introduction

Healthy aging is a multidimensional concept that encompasses the absence of disease and the ability to maintain functional and social well-being in later life. The World Health Organization (WHO) has proposed a capabilities-based approach to measure healthy aging, which focuses on the intrinsic capacity (IC) of individuals as the composite of all the physical and mental capacities that they can draw on at any point in time [1,2]. IC reflects the residual biological potential of the organism rather than its impairments or deficits. It is influenced by both individual and contextual factors that enable people to be and do what they value as they age [3]. IC has been validated and tested as a reliable indicator of healthy aging in different populations and settings, and it has been shown to have predictive value for mortality, disability, and quality of life outcomes [4,5]. IC also provides a holistic and comprehensive framework for assessing and managing the health needs of older people, shifting from a disease-oriented to a function-and-life–oriented approach [6]. However, despite the growing recognition and adoption of IC in clinical and research practice [3,6,7], there is still a lack of understanding of the factors that influence its development and maintenance over time.

One of the potential factors that may affect IC is internet use, which has become increasingly prevalent among older adults in recent years [8]. Internet technology offers various opportunities for enhancing the health and well-being of older people, such as providing access to information, education, entertainment, social support, and health care services [9,10]. For example, a longitudinal study conducted in China found that daily internet use was associated with a lower risk of developing chronic diseases among middle-aged and older adults [11]. However, internet use may also have negative consequences for the health of older people, such as increasing the risk of internet addiction, depression, cognitive decline, sleep disturbance, social isolation, and visual impairment [12-14]. Therefore, the relationship between internet use and healthy aging is complex. It may depend on various factors, such as the frequency, duration, purpose, and quality of internet use [15]. To effectively respond to the challenges of aging, there is a need for a more comprehensive and integrated assessment of the impact of internet use on healthy aging using the IC framework. This would allow us to examine whether internet use can enhance or impair the physical and mental capacities of older people across different domains and dimensions.

This study leveraged data from a representative cohort of individuals aged 45 and older across China to investigate the relationship between daily internet use and IC, a multifaceted indicator of healthy aging. Specifically, the research aims to delineate how varying patterns of internet engagement influence the physical and mental capacities of this demographic, addressing demographic factors and multiple dimensions of activity. By examining these associations, the study seeks to clarify the potential benefits and drawbacks of internet use in preserving or enhancing IC among middle-aged and older adults.

## Methods

### Data and Study Participants

We used publicly available deidentified data from the China Health and Retirement Longitudinal Study (CHARLS) to measure the IC construct and to examine daily internet use and frequency within it. The longitudinal study aims to collect a high-quality, nationally representative sample of Chinese residents ages 45 and older to serve the needs of scientific research on older adults. The baseline national wave of CHARLS was fielded in 2011 and included about 150 counties/districts and 450 villages/resident committees. The individuals were followed up every 2 years [16]. All living respondents from the first and third waves (2011 and 2015) were invited to participate in the physical examinations, blood tests, and life history surveys, which included a series of questions about health and health care history, residential history, education history, and important life events. We included all individuals who participated in the 2011 and 2013 waves and answered the questions about daily internet use. The study began by identifying individuals who had registered at least one instance of internet use during the baseline period. The inclusion criteria required participants to have follow-up records available for the subsequent data collection wave. Those without follow-up data were subsequently excluded. Furthermore, participants were excluded if they had substantial missing data, defined as exceeding 40% missing information, in the IC evaluations conducted. Detailed processes are shown in Figure S1 in Multimedia Appendix 1.

### Daily Internet Use and Frequency

The participants were asked if they had used the internet in the previous month (yes=1, no=0). If the participant’s answer was “yes,” they were asked about their frequency of using the internet in the past month (almost every day=3, almost every week=2, and not often=1) with higher scores indicating higher frequency. In this study, “internet use” refers to daily internet use, which to some extent conforms to the usage habits of middle-aged and older people, including but not limited to instant chatting, viewing news, watching movies, playing games, managing finances, etc [11,17].

### Intrinsic Capacity

The IC used in this cohort study has been proven to have prognostic value in previous studies [3]. IC is an overarching domain containing 5 subdomains, namely locomotion, sensory, vitality, psychological capacity, and cognitive capacity. Each subdomain is assessed by various indicators, such as (1) locomotion: balance, walking speed time, and chair-stand test; (2) sensory: vision and hearing impairments; (3) vitality: forced expiratory volume, grip strength, and hemoglobin; (4) psychological capacity: affect the status, sleep quantity, and quality; (5) cognitive capacity: episodic memory test. The details of the measurement methods are provided in the Supplementary Methods sections 1-10 in Multimedia Appendix 2.

**Figure 1:**
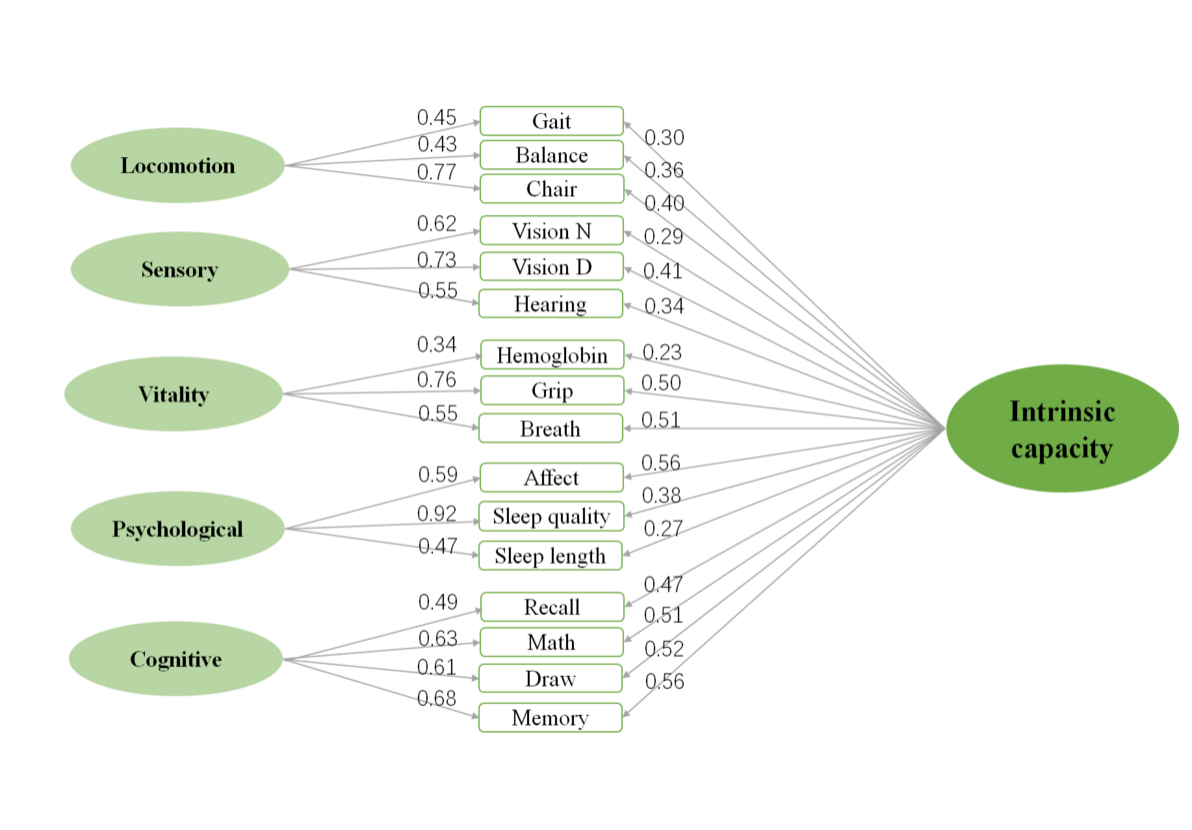
Confirmatory factor analysis bifactor model for intrinsic capacity, illustrating the structural equation modeling used to assess intrinsic capacity with 1 general factor and 5 subfactors.

The score of IC was estimated by a structural equation model (SEM) that included a confirmatory factor analysis (CFA). The CFA was based on the subfactors identified from a previous exploratory factor analysis (EFA). A bifactor model (1 general factor and 6 subfactors) was fitted, which is presented in Figure 1. The goodness of fit statistics, such as root-mean-square error of approximation (RMSEA), comparative fit index (CFI), and Tucker-Lewis index (TLI), were used to test the model fit. The IC score was standardized by subtracting the mean and dividing by the SD. A higher score indicates a stronger capacity to perform valued activities.

### Covariates

The covariates include the basic demographic characteristics, lifestyle behaviors, and health status of the population collected in 2011. Demographic variables were collected, which included age (years), sex (male/female), education level (primary school or lower/middle school/high school or higher), residence (urban/rural), and annual household income. Lifestyle behaviors were obtained from a self-report questionnaire that included the physical activity level (metabolic equivalent physical activities [MET-PA]) [18], social participation monthly frequency (unsocial/occasional/usual), smoking (yes/no), and drinking (yes/no). We explored the potential impact of self-reported health conditions on IC. Specifically, we assessed the correlation between IC scores and 14 self-reported chronic diseases using a Spearman correlation matrix. This approach was chosen under the premise that the accumulation of chronic health conditions may exert an influence on IC. Details of this correlation analysis can be found in Figures S1-S2 in Multimedia Appendix 1. Health conditions contained 14 medically diagnosed conditions (yes/no), including hypertension, dyslipidemia, diabetes, cancer, chronic lung diseases, liver disease, heart diseases, stroke, kidney diseases, digestive diseases, psychiatric problems, memory-related diseases, arthritis or rheumatism, and asthma. CHARLS medical physical examinations recorded BMI, calculated as measured weight (kg) divided by the square of height (m^2^).

### Descriptive Statistics

The baseline characteristics of participants were divided into 2 groups based on whether they used the internet daily. Continuous variables are displayed as mean (SD), and categorical variables are displayed as numbers (percentages). The multiple imputation method was used to fill in the missing data, thereby reducing bias and enhancing the efficiency of our analyses.

### Linear and Restricted Cubic Spline Regression

Multivariate linear regression models were used to evaluate the association of daily internet use and frequency with IC, adjusting for current healthy conditions, as well as demographic, socioeconomic, and lifestyle information. The association we represent is measured as marginal effects on the average of each covariate. The marginal effect and 95% CIs were determined in 5 subdomains. Our main strategy is to use the comprehensive information in survey data to directly explain as many potential confounding factors as possible, as the estimation relationship between IC and benefit factors may deviate when the outcome variable is associated with the error term [19]. Restricted cubic spline (RCS) regression was used to determine the frequency-response association between the frequency of daily internet use and outcomes among IC.

### Subgroup and Sensitivity Analyses

To explore the relationship between daily internet use and IC more comprehensively and reliably, we undertook both subgroup and sensitivity analyses to affirm our findings. The subgroup analysis stratified participants by sex (male/female), age (above/below 60 years), residence (urban/rural), and whether their IC was above or below the median in 2011. Our sensitivity analysis encompassed several steps to ensure robustness: (1) we applied the Mills ratio to account for any increase or decrease in IC total scores from 2011 to 2015; (2) we assessed the consistency of internet usage patterns by designating participants as “constant users” if they reported internet use in both survey waves and as “new users” if they commenced internet use in the 2013 wave after not using it in 2011; (3) data points with extreme IC values were excluded to enhance the stability of our analysis; (4) participants with baseline cognitive impairments, which could potentially affect cognitive subdomain outcomes, such as those with Alzheimer disease, brain atrophy, or Parkinson disease, were omitted from the study; (5) we made adjustments for lifestyle variables using the baseline data from 2015 to account for changes over time; and finally (6) propensity score matching was used to align the baseline characteristics of the study groups, promoting a balanced comparison.

### Mediation Effect Analysis

To investigate whether the impact of IC with its 5 subdomains is mediated by various factors, our study explores the mediating effects of social participation, MET-PA, and chronic diseases on the relationship between daily internet use and IC. The analysis involves several steps. First, we calculate the linear relationship between social participation, MET-PA, chronic diseases, and IC. Second, we use a linear model to analyze the time-related results and all the variables. Finally, we determine the mediation proportion using Preacher and Hayes method [20]. To establish the 95% CIs, we use the bootstrap method, repeating the process 5000 times.

### Statistics Programs

All analyses were conducted using R (version 3.6.1; R Foundation for Statistical Computing) and Stata MP 17 (StataCorp LLC). Cubic spline analysis was performed with the “rcs” package. Subgroup analysis adopted the “Publish” package. The mediation effect was used in the “mediation” package. Multiple imputation method was performed using the “mice” package. Propensity score matching was performed using the “MatchIt” package. A 2-sided *P*<.05 was considered statistically significant.

### Ethical Considerations

CHARLS was a survey approved by the Ethical Review Committee of Peking University (approval number IRB00001052–11015), and the study data were anonymous. Each participant provided signed informed consent at the time of participation. There was no requirement for additional ethics approval for approved data users.

## Result

### Daily Internet Use and Study Population Characteristics

In our study, a total of 12,826 participants were included, with the vast majority (12,305, 95.9%) not using the internet, while 521 (4.1%) individuals reported daily internet use. Among the 521 internet users, 294 (56.4%) were men, and 227 (43.6%) were women, with a mean age of 52.62 years. Further details on the characteristics of the study population can be found in Table 1. Participants who reported daily internet use were more likely to have higher social participation, attain a higher education level, reside in urban areas, and have higher BMI. In addition, this group tended to be younger, predominantly male, and more likely to come from households with higher economic income. In the EFA, 5 factors show eigenvalues greater than 1 (3.69, 1.69, 1.34, 1.25, 1.11). *χ*^2^_104_=32730.621, RMSEA=0.055 (90% CI 0.053-0.058), CFI=0.96, and TLI=0.95 for the 5-factor model and *χ*^2^_75_=347.4, RMSEA=0.102 (90% CI 0.101-0.103), CFI=0.77, and TLI=0.72 for the bifactor model, which suggested the model fits the data well.

**Table 1:** Characteristics of participants (N=12,826) by internet use.

Characteristics and variables^a^			Internet nonuse (n=12,305)	Internet use (n=521)	*P* value	
**Locomotion, mean (SD)**						
	Walking-speed time		1.42 (0.46)	1.32 (0.62)	<.001	
	Balance		0.26 (0.51)	0.10 (0.36)	<.001	
	Chair		2.36 (0.50)	2.16 (0.39)	<.001	
**Sensory, n (%)**						
	**Hearing**					<.001
		Good	1773 (14.4)	139 (26.7)		
		Fair	3709 (30.1)	197 (37.8)		
		Poor	6823 (55.4)	185 (35.5)		
	**Vision distance**					<.001
		Good	1633 (13.3)	147 (28.2)		
		Fair	3032 (24.6)	156 (29.9)		
		Poor	7640 (62.1)	218 (41.8)		
	**Vision near**					<.001
		Good	1178 (9.5)	95 (18.3)		
		Fair	2880 (23.4)	158 (30.3)		
		Poor	8247 (67.0)	268 (51.4)		
**Vitality, mean (SD)**						
	Breath		291.68 (125.86)	385.28 (138.89)	<.001	
	Grip		32.67 (12.30)	39.27 (10.65)	<.001	
	Hemoglobin		14.36 (2.17)	14.51 (1.92)	.12	
**Psychological capacity**						
	Affect, mean (SD)		21.24 (6.48)	24.89 (4.69)	<.001	
	Sleep length, mean (SD)		6.84 (2.15)	7.22 (1.58)	<.001	
	**Sleep quality, n (%)**					<.001
		Good	4293 (34.9)	115 (22.1)		
		Fair	2081 (16.9)	88 (16.9)		
		Poor	5931 (48.2)	318 (61.0)		
**Cognitive capacity**						
	Draw, complete, n (%)		7664 (62.3)	482 (92.5)	<.001	
	Memory, mean (SD)		3.69 (1.42)	4.75 (0.53)	<.001	
	Recall, mean (SD)		6.94 (3.36)	9.93 (3.21)	<.001	
	Math, mean (SD)		2.75 (2.00)	4.04 (1.54)	<.001	
Age (years), mean (SD)			58.50 (9.19)	52.62 (7.67)	<.001	
**Sex^b^, n (%)**						<.001
	Male		5649 (45.9)	294 (56.4)		
	Female		6656 (54.1)	227 (43.6)		
**Residence, n (%)**						<.001
	Urban		2059 (16.7)	390 (74.9)		
	Rural		10,246 (83.3)	131 (25.1)		
BMI (kg/m^2^), mean (SD)			23.48 (3.58)	24.59 (3.40)	<.001	
**Education level, n (%)**						<.001
	Primary school or lower		8658 (70.4)	48 (9.2)		
	Middle school		2536 (20.6)	154 (29.6)		
	High school or higher		1111 (8.9)	319 (61.3)		
**Smoking, n (%)**						.15
	Yes		4706 (38.2)	216 (41.5)		
	No		7599 (61.8)	305 (58.5)		
**Drinking, n (%)**						<.001
	Yes		3978 (32.3)	222 (42.6)		
	No		8327 (67.7)	299 (57.4)		
Household income (US $^c^), mean (SD)			1064.06 (1250.30)	1231.47 (1209.11)	.003	
**Physical activities^d^, n (%)**						
	Vigorous activities		4431 (36.0)	51 (9.8)	<.001	
	Middle activities		7527 (61.2)	280 (53.7)	.001	
	Leisure activities		10,479 (85.2)	463 (88.9)	.02	
**Social participation, n (%)**						<.001
	Unsocial, 0 per/month		6265 (50.9)	157 (30.1)		
	Occasional, 1-3 per/month		5979 (48.6)	324 (62.2)		
	Usual, 4-8 per/month		61 (0.5)	40 (7.7)		
**Chronic disease, n (%)**						
	**Hypertension**					.92
		Yes	2869 (23.3)	123 (23.6)		
		No	9436 (76.7)	398 (76.4)		
	**Dyslipidemia**					<.001
		Yes	1096 (8.9)	93 (17.9)		
		No	11,209 (91.1)	428 (82.1)		
	**Diabetes**					>.99
		Yes	667 (5.4)	28 (5.4)		
		No	11,638 (94.6)	493 (94.6)		
	**Cancer**					.48
		Yes	121 (1.0)	3 (0.6)		
		No	12,184 (99.0)	518 (99.4)		
	**Chronic lung diseases**					.003
		Yes	1228 (10.0)	31 (6.0)		
		No	11,077 (90.0)	490 (94.0)		
	**Liver diseases**					.82
		Yes	483 (3.9)	22 (4.2)		
		No	11,822 (96.1)	499 (95.8)		
	**Heart diseases**					.62
		Yes	1435 (11.7)	65 (12.5)		
		No	10,870 (88.3)	435 (87.5)		
	**Stroke**					>.99
		Yes	226 (1.8)	10 (1.9)		
		No	12,079 (98.2)	511 (98.1)		
	**Kidney diseases**					.73
		Yes	767 (6.2)	30 (5.8)		
		No	11,538 (93.8)	491 (94.2)		
	**Digestive diseases**					.01
		Yes	2908 (23.6)	95 (18.2)		
		No	9397 (76.4)	426 (81.8)		
	**Psychiatric problems**					.17
		Yes	139 (1.1)	2 (0.4)		
		No	12,166 (98.9)	519 (99.6)		
	**Memory-related diseases**					.17
		Yes	138 (1.1)	2 (0.4)		
		No	12,167 (98.9)	519 (99.6)		
	**Arthritis or rheumatism**					<.001
		Yes	4357 (35.4)	92 (17.7)		
		No	7948 (64.6)	429 (82.3)		
	**Asthma**					.05
		Yes	419 (3.4)	9 (1.7)		
		No	11,886 (96.6)	512 (98.3)		

^a^Inconsistencies arise in some values due to rounding.

^b^The interviewer recorded the respondent’s sex as either female or male.

^c^The study used the baseline year (2011 exchange rate of 1 US $=6.4588 CNY for conversion.

^d^Physical activity was defined according to each cohort’s questionnaire. Vigorous activities: Activities that significantly increase heart rate and respiration, such as heavy lifting, digging, aerobics, or fast bicycling. Moderate physical activities: Activities that moderately increase heart rate and respiration, including carrying light loads, regular pace bicycling, or mopping. Leisure activities: These include walking for travel or recreation and other forms of walking for sport or exercise, both at work and at home.

### Association of Daily Internet Use With IC

**Figure 2:**
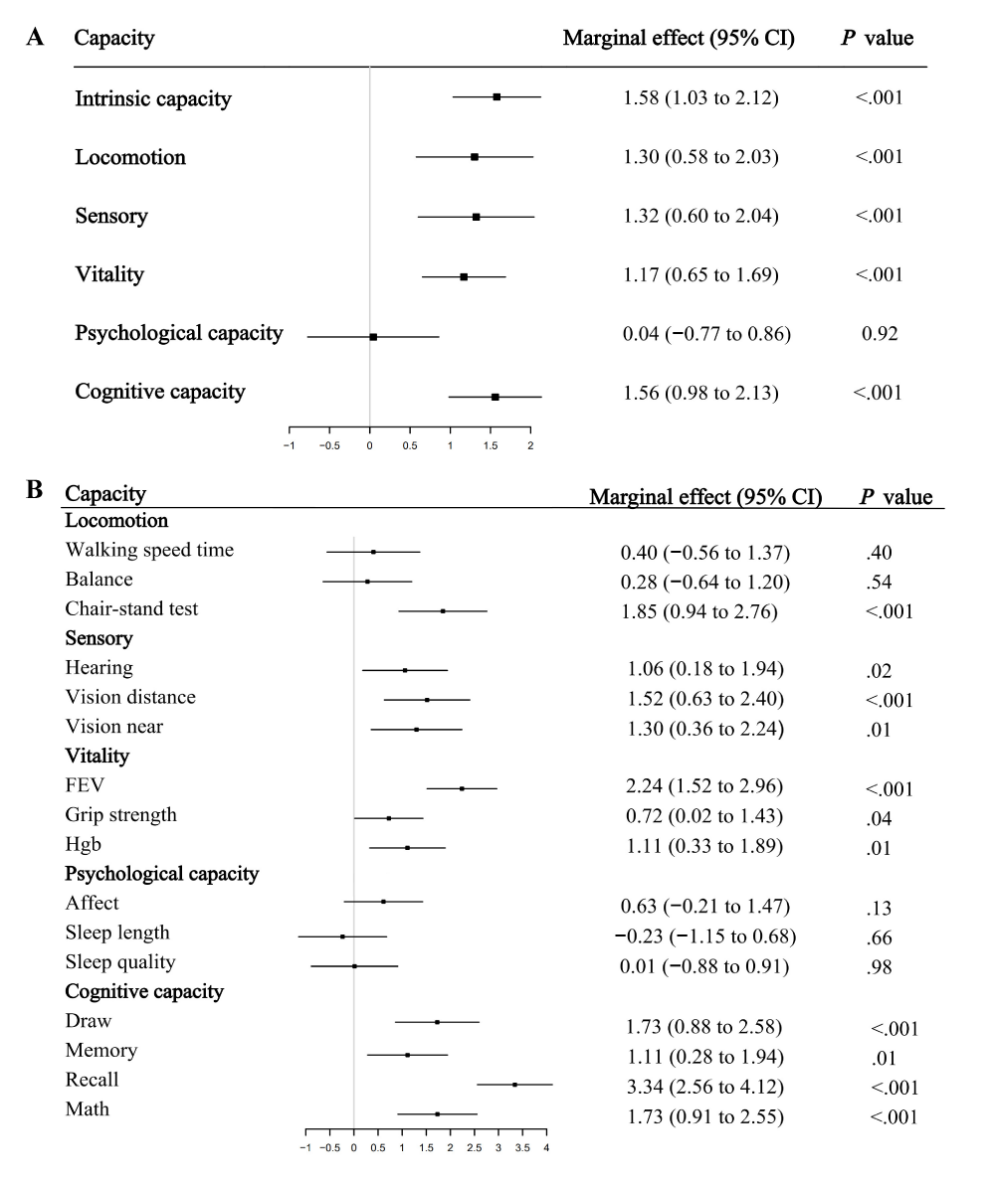
Associations between daily internet use and changes in intrinsic capacity and its subdomains on China Health and Retirement Longitudinal Study (CHARLS) data from 2011 to 2015. FEV: forced expiratory volume; Hgb: hemoglobin.

**Figure 3:**
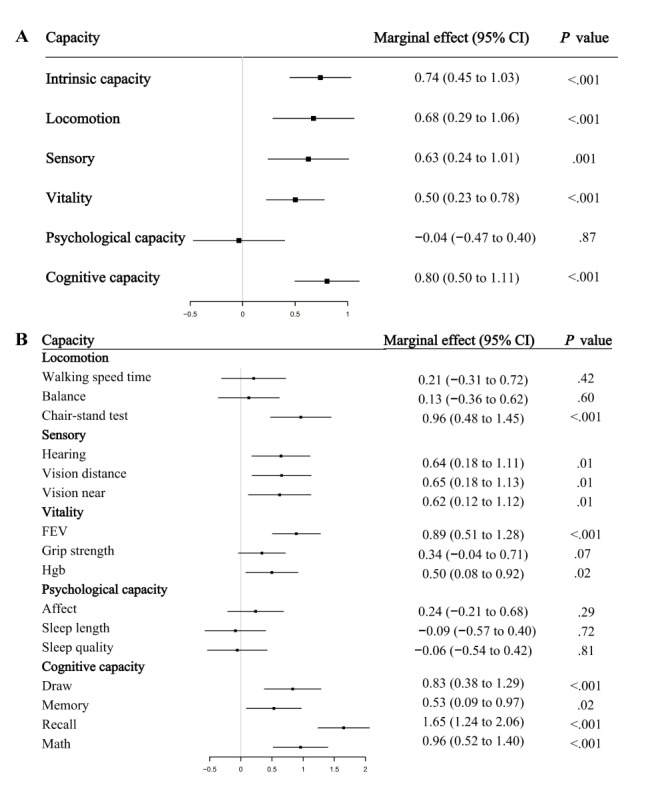
Associations between internet use frequency and changes in intrinsic capacity and its subdomains on China Health and Retirement Longitudinal Study (CHARLS) data from 2011 to 2015. FEV: forced expiratory volume; Hgb: hemoglobin.

In the linear regression analysis, we controlled for demographic variables, socioeconomic information, lifestyle behaviors, and health conditions and examined the effects of daily internet use and frequency on IC change. Figure 2 showed that daily internet use was significantly associated with a slower decline of IC over time (marginal effect 1.58, 95% CI 1.03-2.12; *P*<.001). In addition, an incremental increase in the frequency of Internet use is associated with significant maintenance of IC, as depicted in Figure 3 (marginal effect 0.74, 95% CI 0.45-1.03; *P*<.001).

We further explored the relationship between internet use and the 5 subdomains of IC, including locomotion, sensory, vitality, psychological capacity, and cognitive capacity. In Figure 2, internet use was positively linked to all subdomains except psychological capacity, with the strong effects on locomotion (marginal effect 1.30, 95% CI 0.58-2.03; *P*<.001), sensory (marginal effect 1.32, 95% CI 0.60-2.04; *P*<.001), vitality (marginal effect 1.17, 95% CI 0.65-1.69; *P*<.001) and cognitive capacity (marginal effect 1.56, 95% CI 0.98-2.13; *P*<.001). In Figure 3, we observed similar patterns when analyzing the effects of internet frequency on the subdomains of IC, with positive associations for all subdomains except psychological capacity and the large effects for locomotion (marginal effect 0.68, 95% CI 0.29-1.06; *P*<.001), sensory (marginal effect 0.63, 95% CI 0.24-1.01; *P*<.001), vitality (marginal effect 0.50, 95% CI 0.23-0.78; *P*<.001), and cognitive capacity (marginal effect 0.80, 95% CI 0.50-1.11; *P*<.001).

### Examination of the Frequency–Response Effect

**Figure 4:**
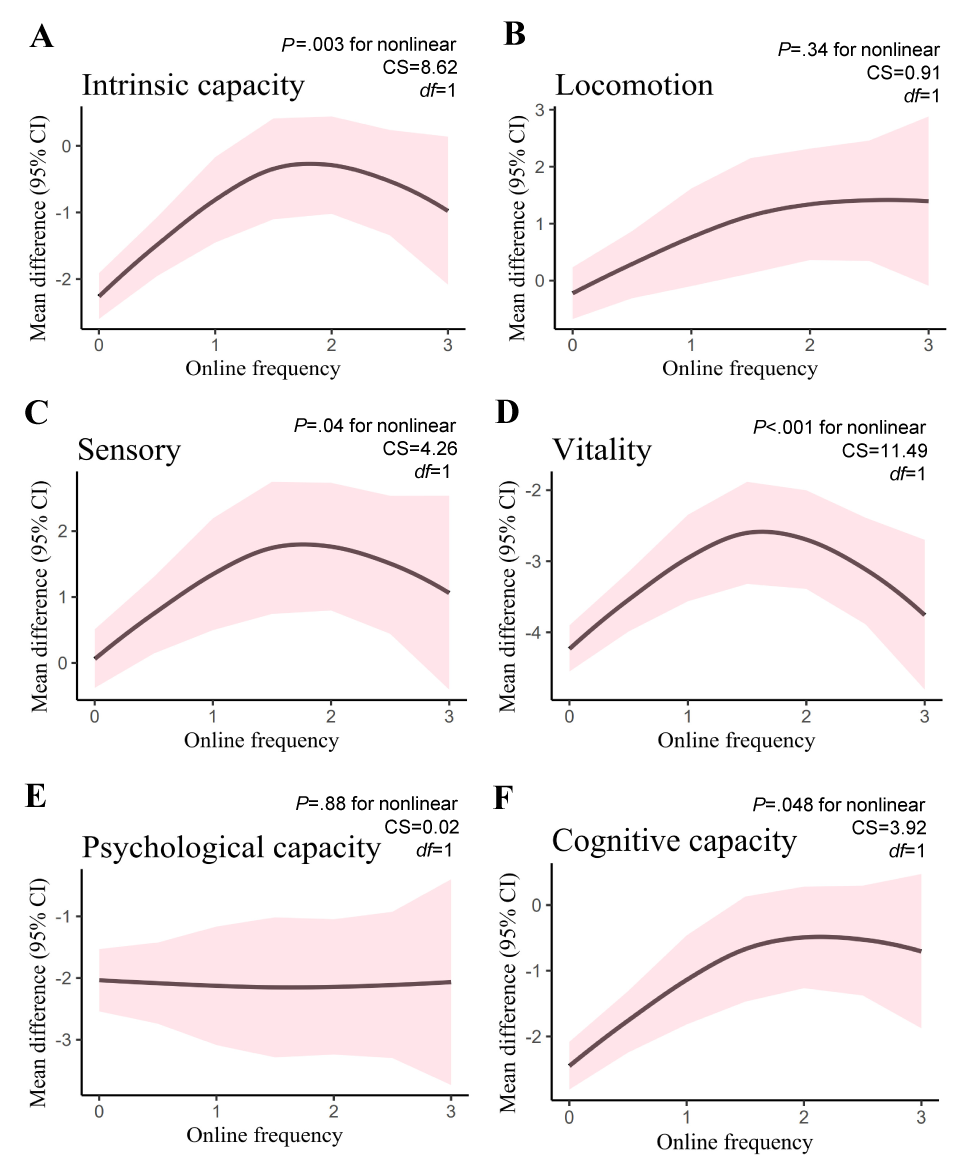
Impact of internet use frequency on intrinsic capacity change (2011-2015). X-axis represents internet usage frequency (almost every day=3, almost every week=2, not often=1, non-use=0), Y-axis represents the mean difference between the 2015 intrinsic capacity score and the 2011 intrinsic capacity score. CS: chi-square; df: degrees of freedom.

We used RCS functions to examine the relationship between IC and its 5 subdomains with daily internet use. Figure 4 shows the results of the frequency-response effect analysis on IC and its subdomains. The analysis revealed a non-linear, inverted U-shaped relationship between IC change and internet use frequency (nonlinear *P*=.003), indicating that there was a positive association within a certain range of internet use frequency but no significant additional benefits beyond that range. Similarly, sensory (nonlinear *P*=.04), vitality (nonlinear *P*<.001), and cognitive capacity (nonlinear *P*=.048) showed positive associations with increasing internet use frequency within a moderate range.

### Subgroup Analyses and Sensitivity Analyses

We performed multiple subgroup analyses and sensitivity analyses to test the robustness of our results, which are shown in Figures S7-S12 in Multimedia Appendix 1. We found that internet use had a differential impact on IC across age groups and genders. Specifically, older individuals (aged 60 years and older) seem to benefit more from internet use in terms of locomotion (*P*=.02 for interaction) and vitality (*P=*.01 for interaction) than younger ones (under 60 years of age). Similarly, women show more improved cognitive capacity (*P*=.03 for interaction) while men enhanced more in vitality (*P*<.001 for interaction) through internet use. Our findings were also stable under various sensitivity analyses and did not materially change our results (Tables S3-S4 in Multimedia Appendix 3)

### Mediation Analysis for Social Participation, Physical Activity, and Chronic Diseases

To further explore the mechanisms underlying the relationship between daily internet use and IC, we conducted a mediation analysis to assess the roles of social participation, physical activity, and chronic diseases (Table S5 in Multimedia Appendix 3). The results revealed that social participation was a significant mediator between daily internet use and IC change, explaining 28.78% of the total effect (95% CI 21.24-40.33; *P*<.001), especially for the domains of locomotion, sensory, vitality, and cognitive capacity. We also confirmed some mediation effects from leisure exercise (4.26%, 95% CI 1.33-9.79; *P*=.002) and arthritis/rheumatic diseases (1.42%, 95% CI 0.30-3.02, *P*=.01).

## Discussion

### Principal Findings

In this prospective cohort study involving middle-aged and older adults, we found an inverted U-shaped association between daily internet use and IC. Specifically, we observed that daily internet use and increased frequency of use were associated with higher levels of cognitive capacity, sensory function, vitality, and locomotion. The results of our study underscore the potential advantages of maintaining a moderate and regular pattern of internet use in promoting and preserving IC.

### Comparison to Previous Studies

Our study assessed the association between daily internet use and IC, extending beyond the focus of previous research on frailty, resilience, and aging [21-23]. IC provides a comprehensive measure of overall health status, facilitating early interventions to prevent significant health deteriorations. Unlike other aging concepts, IC emphasizes functional abilities rather than the mere presence or absence of disease [2,24]. It also considers personal attributes, environmental factors, and their interactions, enabling cross-time, cross-country, and cross-cultural comparisons of healthy aging [1,25,26].

The global interest in the association between daily internet use and cognitive capacity has been growing. Functional imaging scans have shown that older adults who were not familiar with the internet exhibited significant increases in brain neural activity when they learned to search online [27]. Certain computer programs and video games have demonstrated the potential to improve memory, multitasking abilities, fluid intelligence, and other cognitive functions [27]. Previous research has also suggested that engaging in brain-challenging activities like online searches may positively affect brain health and possibly delay cognitive decline [28]. The findings of our study supported these results by assessing various cognitive tasks, such as math, drawing, memory, and delayed recall. Furthermore, another study indicates that internet use may act as a protective factor against mild cognitive impairment in Chinese older individuals, potentially mitigating cognitive decline by influencing the volume of the globus pallidus [29]. The association between daily internet use and vitality is also consistent. This might be explained by the use of social media to promote body image management and engage in muscle exercise, which may stimulate physical vitality [30]. Previous hypotheses proposed that vitality is a biological change inherent to aging and serves as a marker of individual physiological resilience [24]. In this context, the daily use of the internet may help to delay age-related biomarkers to some extent [11]. In addition, most individuals who use the internet daily tend to have good hearing and vision, and middle-aged and older individuals who use the internet often experience stimulation through visual and auditory senses [31]. Therefore, the benefits of internet use among this population are receiving increasing attention [8]. The relationship between daily internet use and locomotion is also noteworthy. A study has highlighted the positive impact of internet use on the physical health of middle-aged and older individuals, with exercise frequency serving as a mediating factor between these variables [32]. It is plausible that accessing health-related information through the internet motivates middle-aged and older individuals to actively participate in exercise and improve their physical fitness [33,34].

The lack of a direct positive association between daily internet use and psychological capacity is somewhat unexpected. Previous research has indeed shown that internet use may reduce symptoms of depression [9,15], yet it also may negatively affect sleep quality [15], which is crucial for psychological well-being. In our study, sleep quality and duration emerged as significant factors influencing psychological capacity, particularly among older adults, who often face sleep-related challenges [35]. We hypothesize that while the internet might offer some psychological benefits, its usage can also lead to poor sleep habits, such as staying up late [36]. This, in turn, could negate potential positive impacts on psychological capacity. Therefore, the absence of a significant positive association in our findings does not necessarily contradict previous literature but highlights the complex interplay of factors affecting psychological outcomes in the aging population [37,38]. This nuanced interpretation is supported by studies suggesting that prolonged internet usage or mood fluctuations induced by internet content may lead to detrimental cycles not conducive to the mental health of older adults with preexisting sleep issues [39].

Heterogeneity analysis revealed that the impact of internet use on IC varies based on age and gender. Internet use had a more significant promoting effect on cognitive capacity in older women compared with men. This may be attributed to the generational context of women in this study, who may have a stronger desire for social resources due to historical gender biases [40]. Women are more likely to maintain positive social networks, obtaining more instrumental and emotional resources that support good cognition and active engagement in life [21,41]. On the other hand, middle-aged and older men who use the internet appear more energetic, possibly because they are more inclined to showcase physical attributes like muscle strength on social media [30]. Older individuals (older than 60 years) benefited more from internet use in terms of locomotion and vitality compared to younger individuals, possibly due to poorer physical conditions and fewer acceptable ways to promote health in the older group [25].

The results of the mediation analysis showed that internet use is positively associated with physical exercise behavior and social participation of older people, as well as arthritis or rheumatic diseases, which is consistent with the results of previous research [11,32,42]. Regarding social participation, some researchers believe that when using the internet to chat with strangers, they tend to stay away from their families and reduce social activities, resulting in dissatisfaction with life [12,43]. A prevailing viewpoint among researchers is that the internet may overcome time and space limitations, thereby increasing opportunities for social connections and promoting meaningful contact with close relatives and friends [44,45]. In addition, internet use may enhance the social adaptability of older adults, helping them to better integrate into a wide range of social groups [46,47].

### Strength and Limitations

The study investigates the relationship between daily internet use and IC based on a national large-scale prospective cohort of middle-aged and older people. However, it also has several limitations that may affect the interpretation and generalization of the results. First, as the database sample consists of Chinese subjects only, this may limit the applicability of the research results to other countries or populations. In addition, the average age of internet-using respondents in our study is 52, indicating a comparatively younger subset of the older adult population. Second, the database used in this study did not collect detailed information on the main modes and features of internet use, as well as the motivations for internet use. Therefore, we could not explore how different types of internet use may affect IC differently. Third, there is no precise measure of usage frequency that can provide a reasonable online duration. We only used self-reported categories of daily internet use, which may introduce measurement error and bias. Fourth, the data reflects usage patterns from a period that may not accurately capture current trends due to the rapid evolution and increasing ubiquity of internet technology over time. There are still many other possible factors that may mediate or mitigate the effect of internet use on IC, which are worth further investigation. For example, social support, personality traits, and lifestyle factors may influence both internet use and IC. Although we controlled for some potential confounders in the analysis, we cannot completely rule out the possibility of residual confounding or reverse causation.

### Policy Recommendations

According to the 48th Statistical Report on the Development of Chinese Internet, China’s Internet penetration rate rose from 45.80% in 2013 to 75.60% in December 2022, and the number of Internet users increased from 618 million in 2013 to 1067 million in 2022 [8]. Notably, older individuals are more likely to use the internet for routine daily activities, such as watching videos, staying informed about current events, and health care delivery [11,48]. Internet use may also provide older people with monitoring of routine functional abilities and depression, representing one way to aid their social integration, increase social participation, and, thus, reduce depressive symptoms [9,42]. The daily use of the internet by older adults has been found to have a significant impact on healthy aging in various aspects. Therefore, the following beneficial policy support can be considered: (1) encouraging moderate internet use: we recommend that older individuals engage in internet activities at a moderate frequency to maximize benefits and avoid the potential drawbacks of excessive use; (2) educational campaigns: it is essential to implement educational and promotional campaigns that inform older adults about healthy internet practices, enhancing their understanding and guiding them towards effective use; and (3) enhancing internet literacy: we propose the expansion of internet literacy training programs designed specifically for older adults to improve their abilities to access information, communicate, and interact online, thus maximizing the benefits of internet connectivity [49]. These policy supports can help older people better enjoy the convenience and benefits brought by the Internet and promote digital inclusion and healthy aging of older adults.

### Conclusions

In conclusion, this study aims to investigate the relationship between daily internet usage and IC, an aspect that has not been previously explored. IC serves as a crucial assessment and predictive system for healthy aging, as it reflects individuals’ functional abilities and overall well-being. The results of our study underscore the potential advantages of maintaining a moderate and regular pattern of internet use in promoting and preserving IC, particularly in terms of cognitive capacity, sensory capacity, vitality, and locomotion. The effect may be related to social participation. Consequently, our results offer valuable insights for developing interventions to promote healthy aging among the middle-aged and older adult population.

## Appendix

Multimedia Appendix 1 Additional figures (S1-S13) for the study.

Multimedia Appendix 2 Additional methods for intrinsic capacity measurement in this study.

Multimedia Appendix 3 Additional tables (S1-S5) for the study.

## Abbreviations

CFA: confirmatory factor analysis
CFI: comparative fit index
CHARLS: China Health and Retirement Longitudinal Study
EFA: exploratory factor analysis
IC: intrinsic capacity
MET-PA: metabolic equivalent physical activities
RCS: restricted cubic spline
RMSEA: root-mean-square error of approximation
SEM: structural equation model
TLI: Tucker-Lewis index
WHO: World Health Organization

## Data Availability

The datasets analyzed during this study are available in the CHARLS repository [50].
