# Supplementary material for: Association Between Daily Internet Use and Intrinsic Capacity Among Middle-Aged and Older Adults in China: Large Prospective Cohort Study
Source: J Med Internet Res. 2024 Nov 12;26:e54200. doi: 10.2196/54200 (PMC11599878; doi:10.2196/54200)
Supplement: Multimedia Appendix 1 [file jmir_v26i1e54200_app1.docx]

**Supplemental Figures 1-11**

**
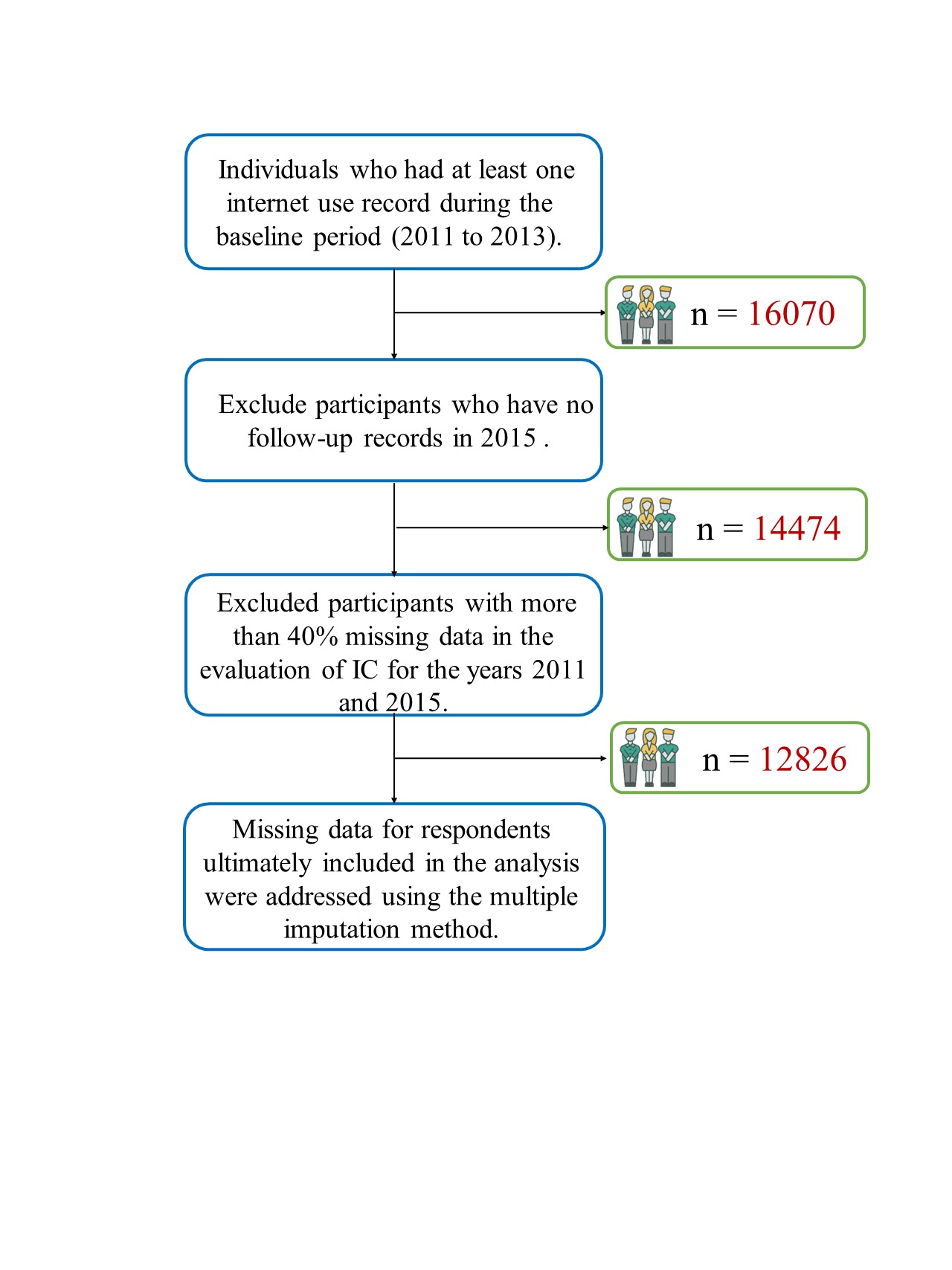
**

**Figure S1.** Flow diagram detailing the inclusion and exclusion criteria for participant selection in the study.

**
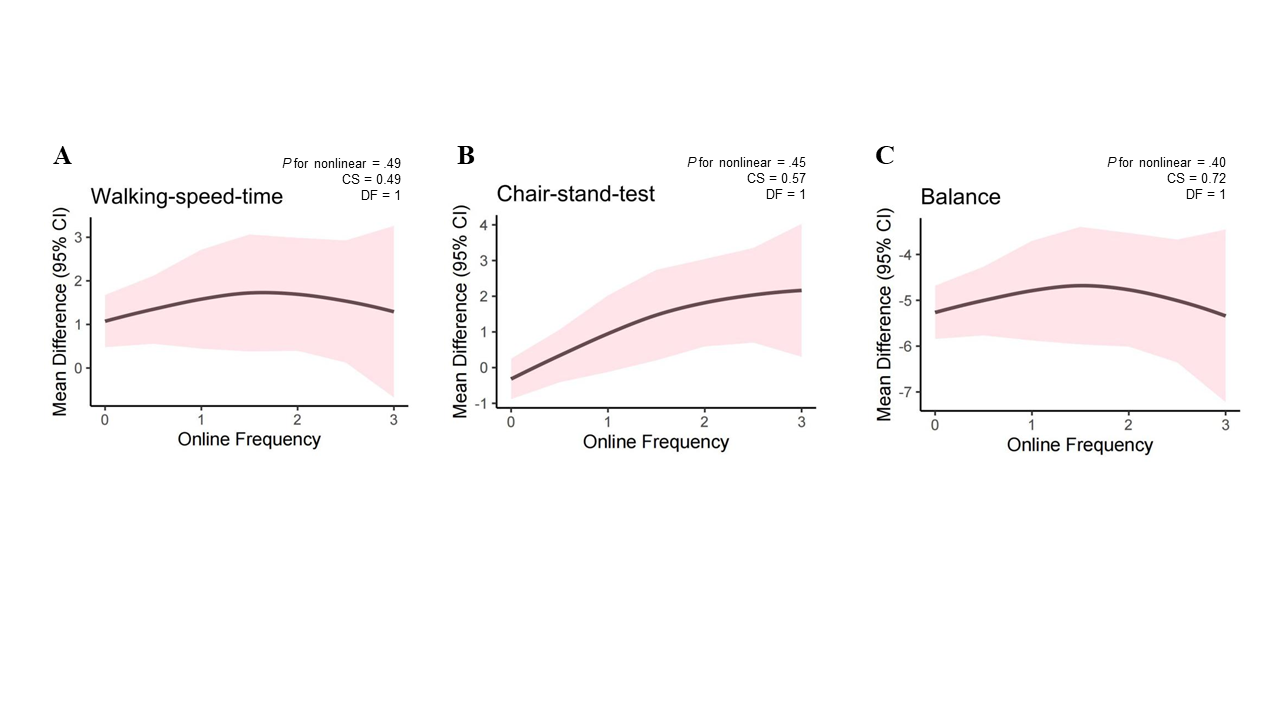
**

**Figure S2.** Association between daily Internet use and locomotion indicators, illustrated through restricted cubic spline models in CHARLS data (2011–2015) for (A) walking speed time, (B) chair-stand test, and (C) balance. The analysis was conducted by adjusting for demographic variables (age, sex, residence, education level, and annual household income) + lifestyle behaviors (drinking history, smoking history, and MET-PA) + health conditions (BMI, hypertension, dyslipidemia, diabetes, cancer, chronic lung diseases, liver disease, heart diseases, stroke, kidney diseases, digestive diseases, psychiatric problems, memory-related diseases, arthritis or rheumatism, and asthma). The value of the X-axis represents Internet use frequency (almost every day=3, almost every week =2, not often=1, non-use=0).

Abbreviations: CS, Chi-Square; DF, degrees of freedom. BMI, body mass index; IC, intrinsic capacity.

**
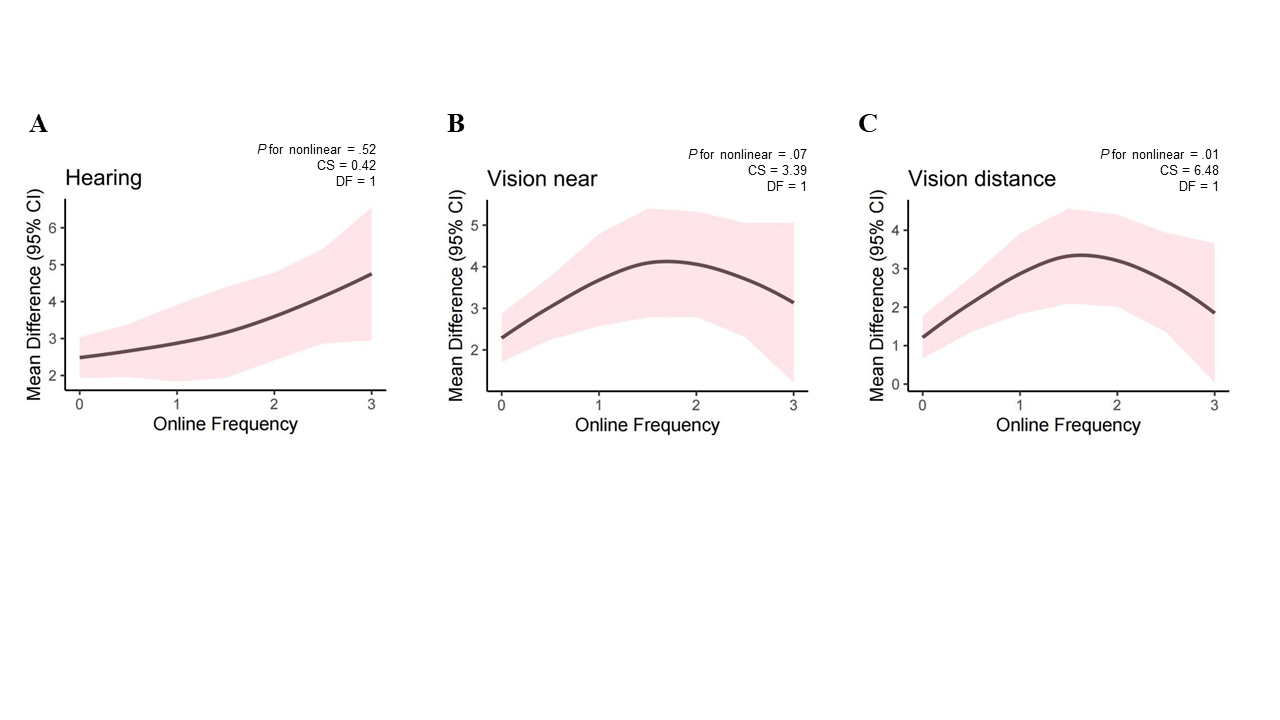
**

**Figure S3.** Association between daily Internet use and sensory indicators, illustrated through restricted cubic spline models in CHARLS data (2011–2015) for (A) walking speed time, (B) chair-stand test, and (C) balance. The analysis was conducted by adjusting for demographic variables (age, sex, residence, education level, and annual household income) + lifestyle behaviors (drinking history, smoking history, and MET-PA) + health conditions (BMI, hypertension, dyslipidemia, diabetes, cancer, chronic lung diseases, liver disease, heart diseases, stroke, kidney diseases, digestive diseases, psychiatric problems, memory-related diseases, arthritis or rheumatism, and asthma). The value of the X-axis represents Internet use frequency (almost every day=3, almost every week =2, not often=1, non-use=0).

Abbreviations: CS, Chi-Square; DF, degrees of freedom. BMI, body mass index; IC, intrinsic capacity.


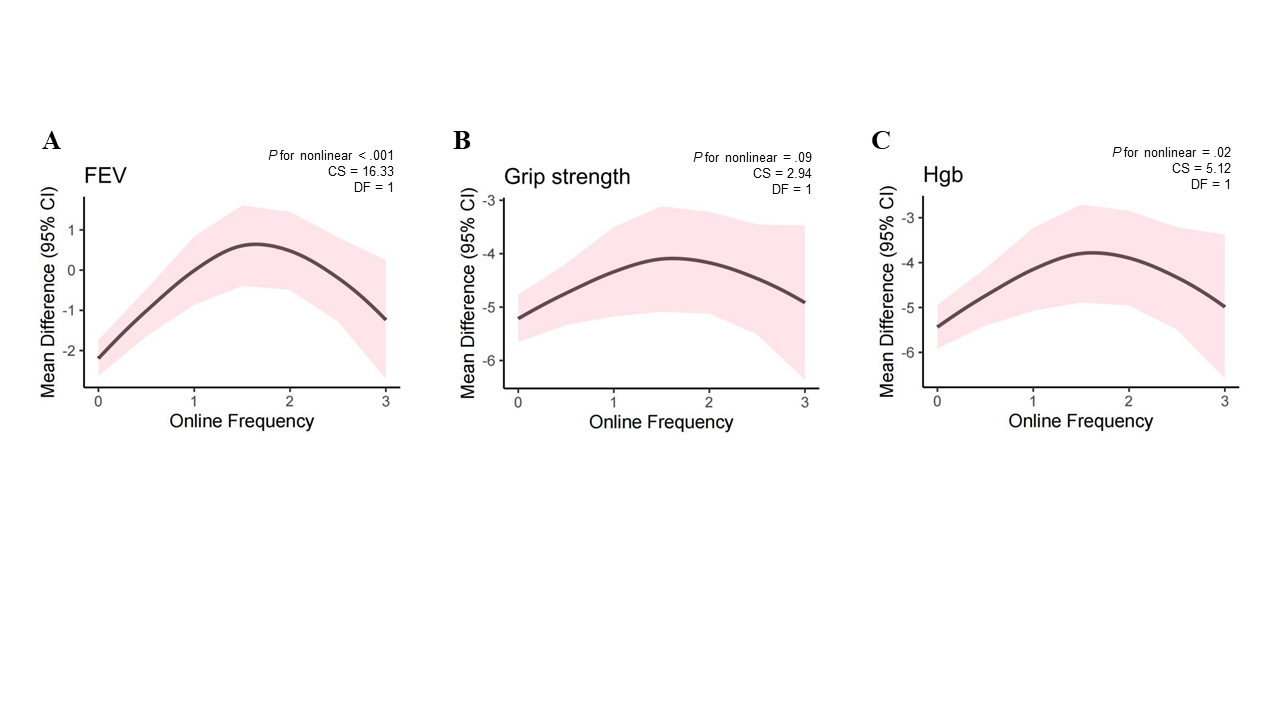


**Figure S4.** Association between daily Internet use and vitality indicators, illustrated through restricted cubic spline models in CHARLS data (2011–2015) for (A) walking speed time, (B) chair-stand test, and (C) balance. The analysis was conducted by adjusting for demographic variables (age, sex, residence, education level, and annual household income) + lifestyle behaviors (drinking history, smoking history, and MET-PA) + health conditions (BMI, hypertension, dyslipidemia, diabetes, cancer, chronic lung diseases, liver disease, heart diseases, stroke, kidney diseases, digestive diseases, psychiatric problems, memory-related diseases, arthritis or rheumatism, and asthma). The value of the X-axis represents Internet use frequency (almost every day=3, almost every week =2, not often=1, non-use=0).

Abbreviations: CS, Chi-Square; DF, degrees of freedom. BMI, body mass index; IC, intrinsic capacity. Hgb, hemoglobin. FEV, Forced breathing volume.


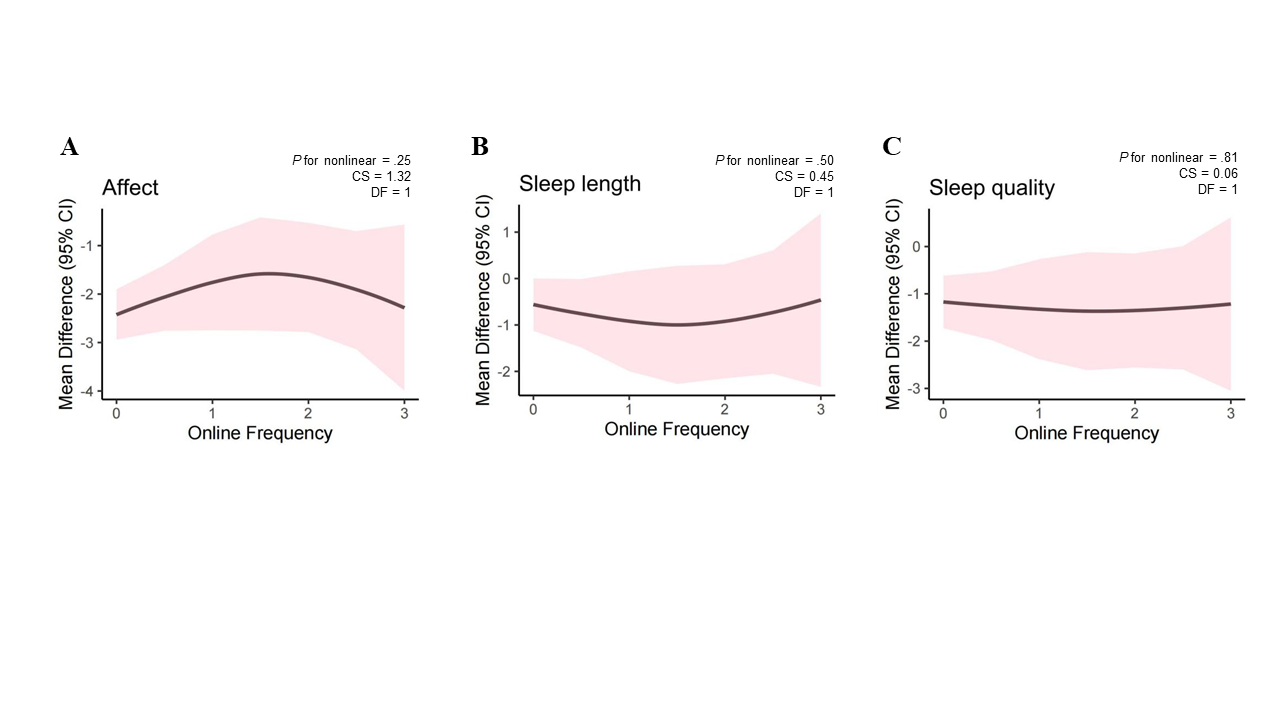


**Figure S5.** Association between daily Internet use and psychological capacity indicators, illustrated through restricted cubic spline models in CHARLS data (2011–2015) for (A) walking speed time, (B) chair-stand test, and (C) balance. The analysis was conducted by adjusting for demographic variables (age, sex, residence, education level, and annual household income) + lifestyle behaviors (drinking history, smoking history, and MET-PA) + health conditions (BMI, hypertension, dyslipidemia, diabetes, cancer, chronic lung diseases, liver disease, heart diseases, stroke, kidney diseases, digestive diseases, psychiatric problems, memory-related diseases, arthritis or rheumatism, and asthma). The value of the X-axis represents Internet use frequency (almost every day=3, almost every week =2, not often=1, non-use=0).

Abbreviations: CS, Chi-Square; DF, degrees of freedom. BMI, body mass index; IC, intrinsic capacity.


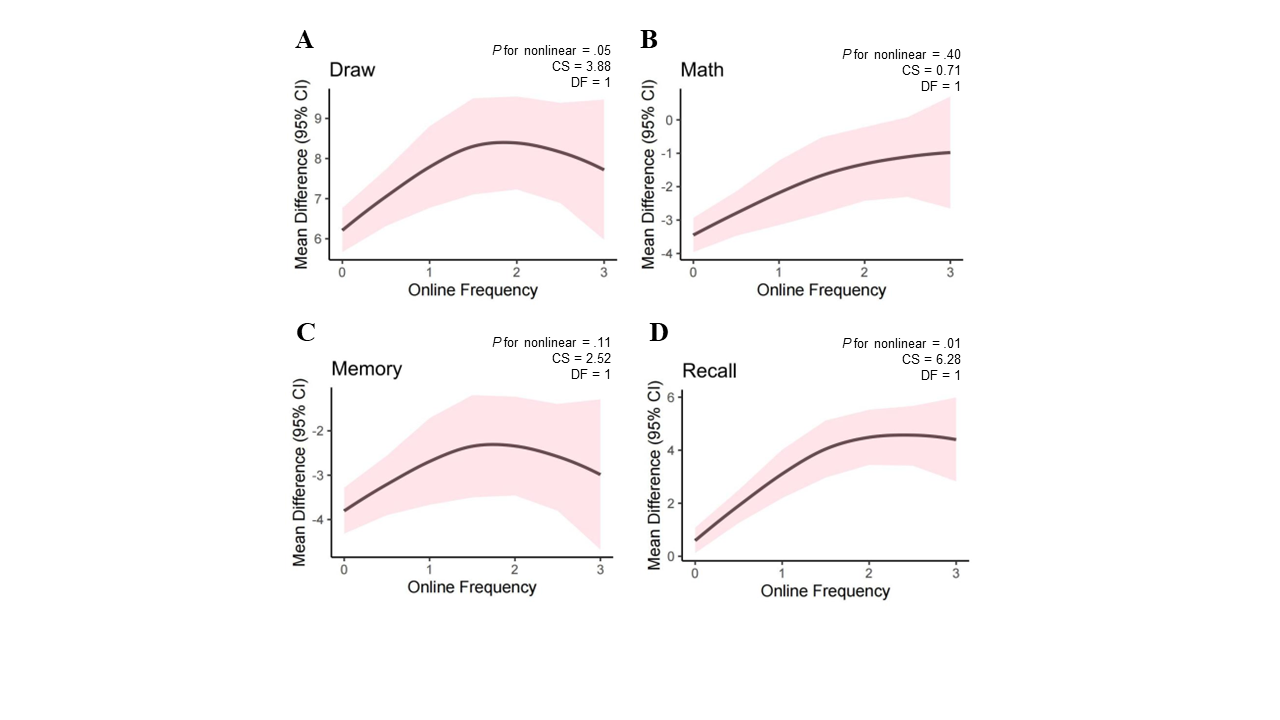


**Figure S6.** Association between daily Internet use and cognitive capacity indicators, illustrated through restricted cubic spline models in CHARLS data (2011–2015) for (A) walking speed time, (B) chair-stand test, and (C) balance. The analysis was conducted by adjusting for demographic variables (age, sex, residence, education level, and annual household income) + lifestyle behaviors (drinking history, smoking history, and MET-PA) + health conditions (BMI, hypertension, dyslipidemia, diabetes, cancer, chronic lung diseases, liver disease, heart diseases, stroke, kidney diseases, digestive diseases, psychiatric problems, memory-related diseases, arthritis or rheumatism, and asthma). The value of the X-axis represents Internet use frequency (almost every day=3, almost every week =2, not often=1, non-use=0).

Abbreviations: CS, Chi-Square; DF, degrees of freedom. BMI, body mass index; IC, intrinsic capacity.


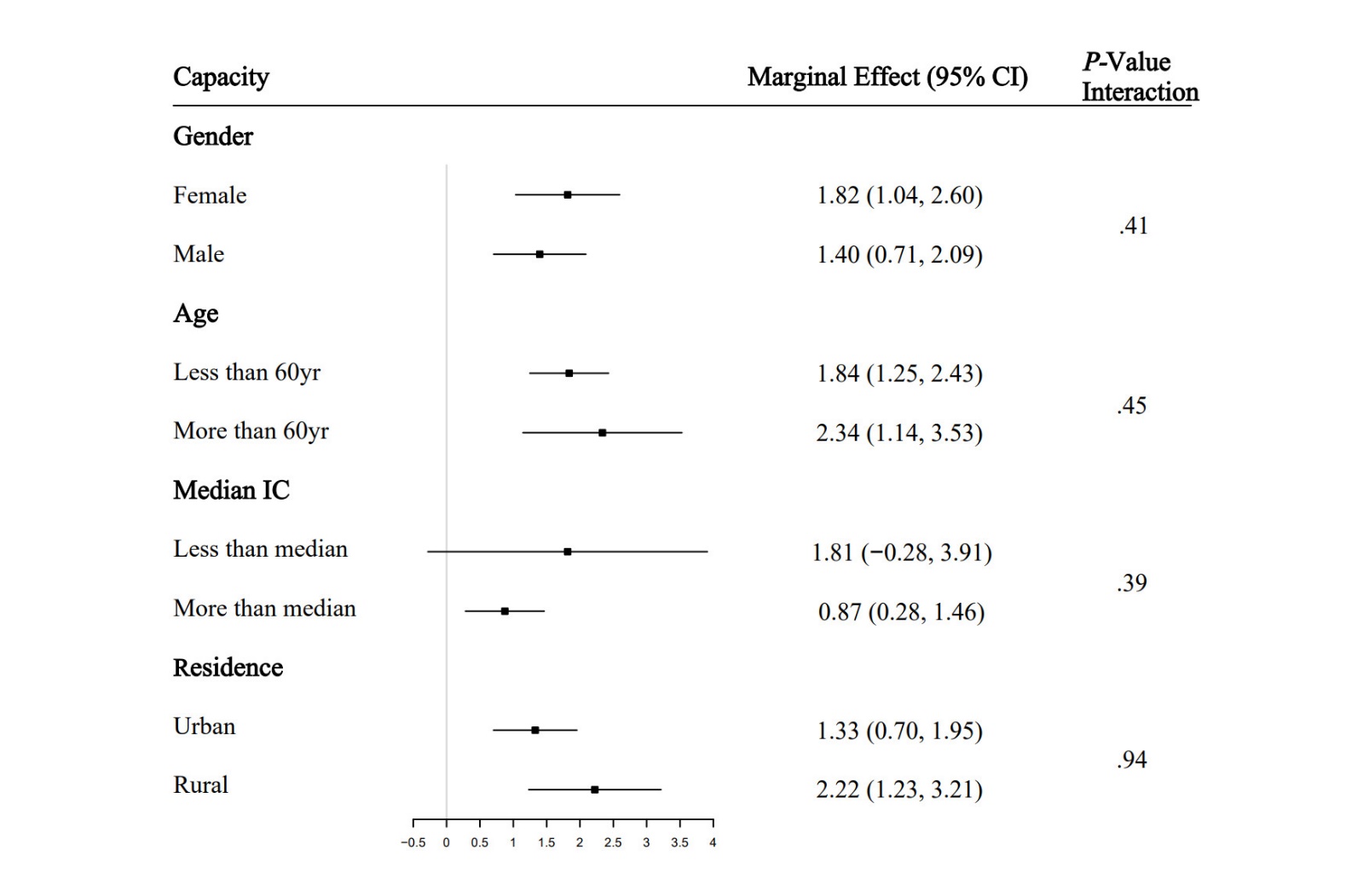


**Figure S7**. Subgroup analyses for the association between the total score of intrinsic capacity and daily internet use by gender, age, residence, and median 2011 IC, adjusted for demographic variables (age, gender, residence, education level and annual household income) +lifestyle (drinking history, smoking history, social participation and MET-PA) + health status (BMI, hypertension, dyslipidemia, diabetes, cancer, chronic lung diseases, liver disease, heart diseases, stroke, kidney diseases, digestive diseases, psychiatric problems, memory-related diseases, arthritis or rheumatism, and asthma). The IC total score was adjusted using the residual method.

Abbreviations: IC, intrinsic capacity; CI, confidence intervals.


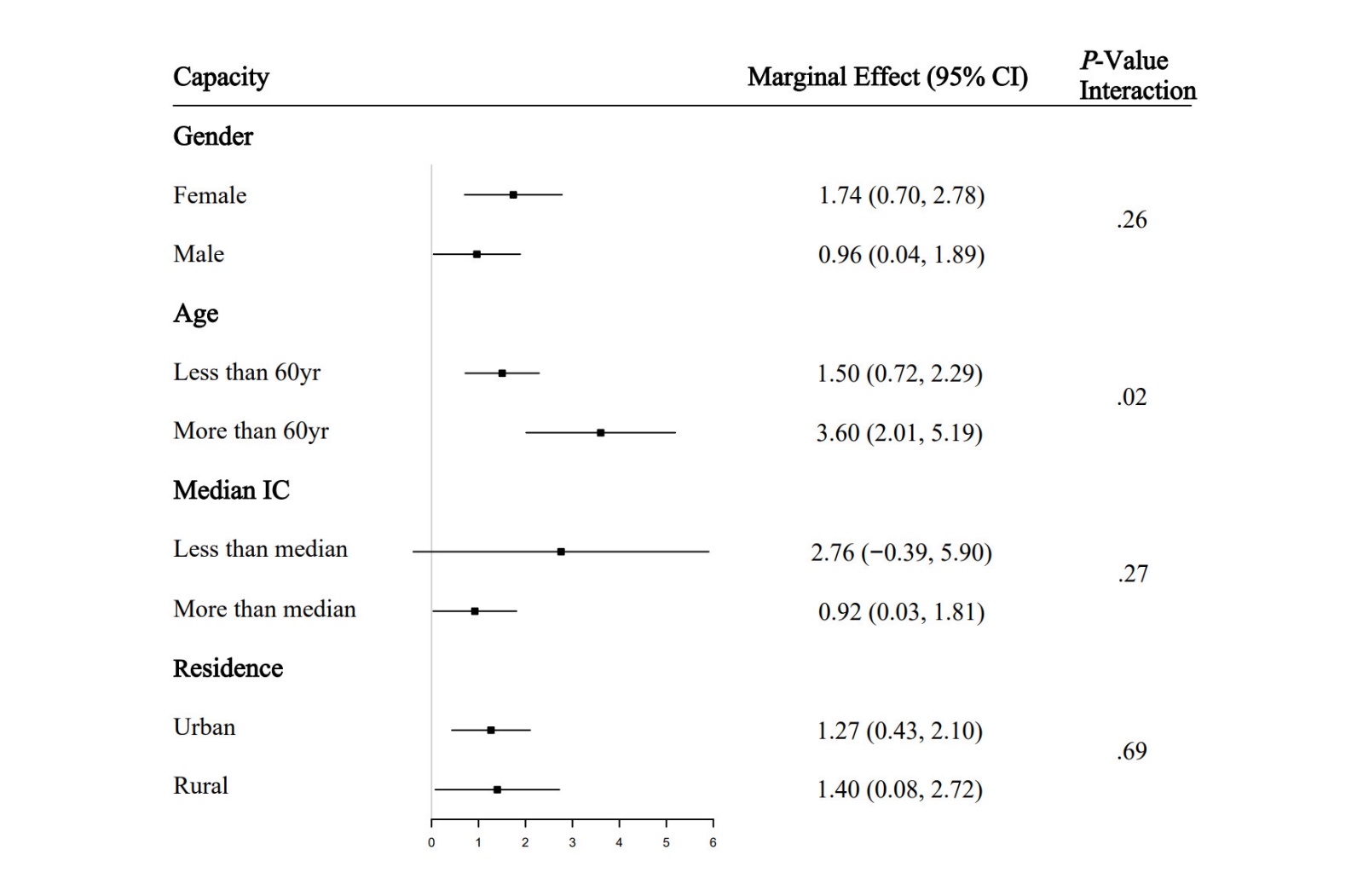


**Figure S8**. Subgroup analyses for the association between the intrinsic capacity with its locomotion and daily internet use by gender, age, residence, and median 2011 IC, adjusted for demographic variables (age, gender, residence, education level and annual household income) +lifestyle (drinking history, smoking history, social participation and MET-PA) + health status (BMI, hypertension, dyslipidemia, diabetes, cancer, chronic lung diseases, liver disease, heart diseases, stroke, kidney diseases, digestive diseases, psychiatric problems, memory-related diseases, arthritis or rheumatism, and asthma). The IC total score was adjusted using the residual method.

Abbreviations: IC, intrinsic capacity; CI, confidence intervals.


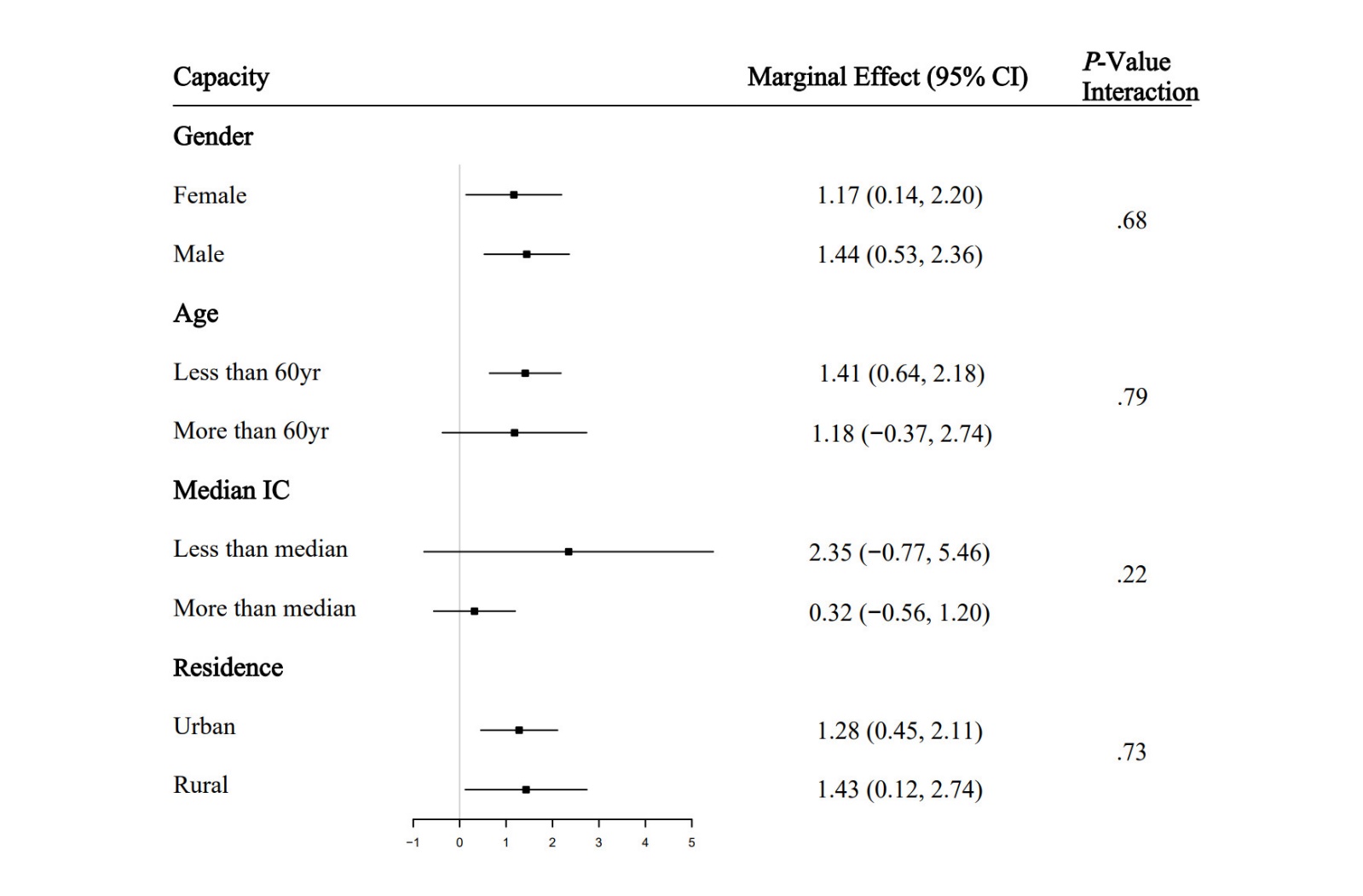


**Figure S9**. Subgroup analyses for the association between the intrinsic capacity with its sensory and daily internet use by gender, age, residence, and median 2011 IC, adjusted for demographic variables (age, gender, residence, education level and annual household income) +lifestyle (drinking history, smoking history, social participation and MET-PA) + health status (BMI, hypertension, dyslipidemia, diabetes, cancer, chronic lung diseases, liver disease, heart diseases, stroke, kidney diseases, digestive diseases, psychiatric problems, memory-related diseases, arthritis or rheumatism, and asthma). The IC total score was adjusted using the residual method.

Abbreviations: IC, intrinsic capacity; CI, confidence intervals.


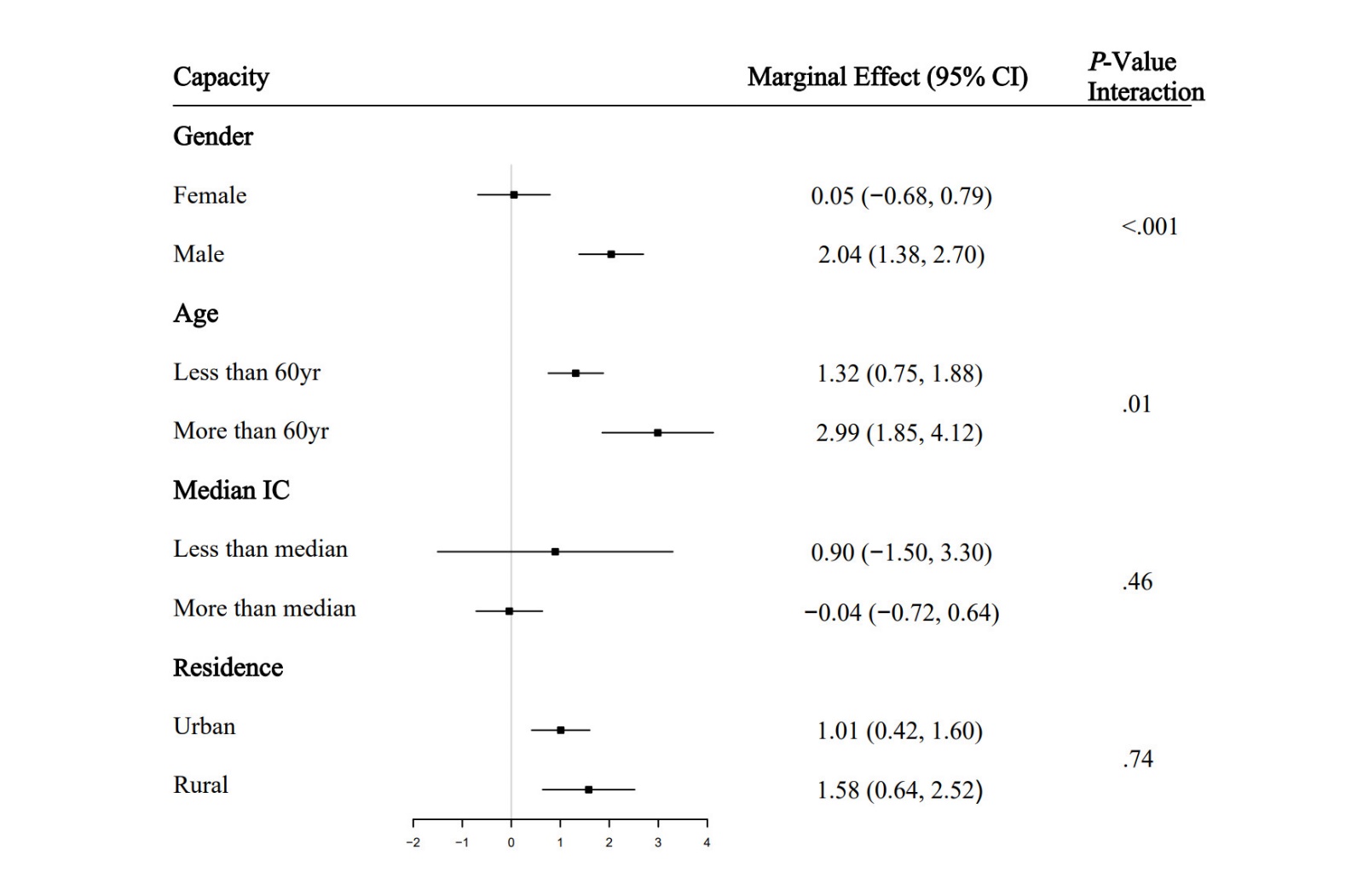


**Figure S10**. Subgroup analyses for the association between the intrinsic capacity with its vitality and daily internet use by gender, age, residence, and median 2011 IC, adjusted for demographic variables (age, gender, residence, education level and annual household income) +lifestyle (drinking history, smoking history, social participation and MET-PA) + health status (BMI, hypertension, dyslipidemia, diabetes, cancer, chronic lung diseases, liver disease, heart diseases, stroke, kidney diseases, digestive diseases, psychiatric problems, memory-related diseases, arthritis or rheumatism, and asthma). The IC total score was adjusted using the residual method.

Abbreviations: IC, intrinsic capacity; CI, confidence intervals.


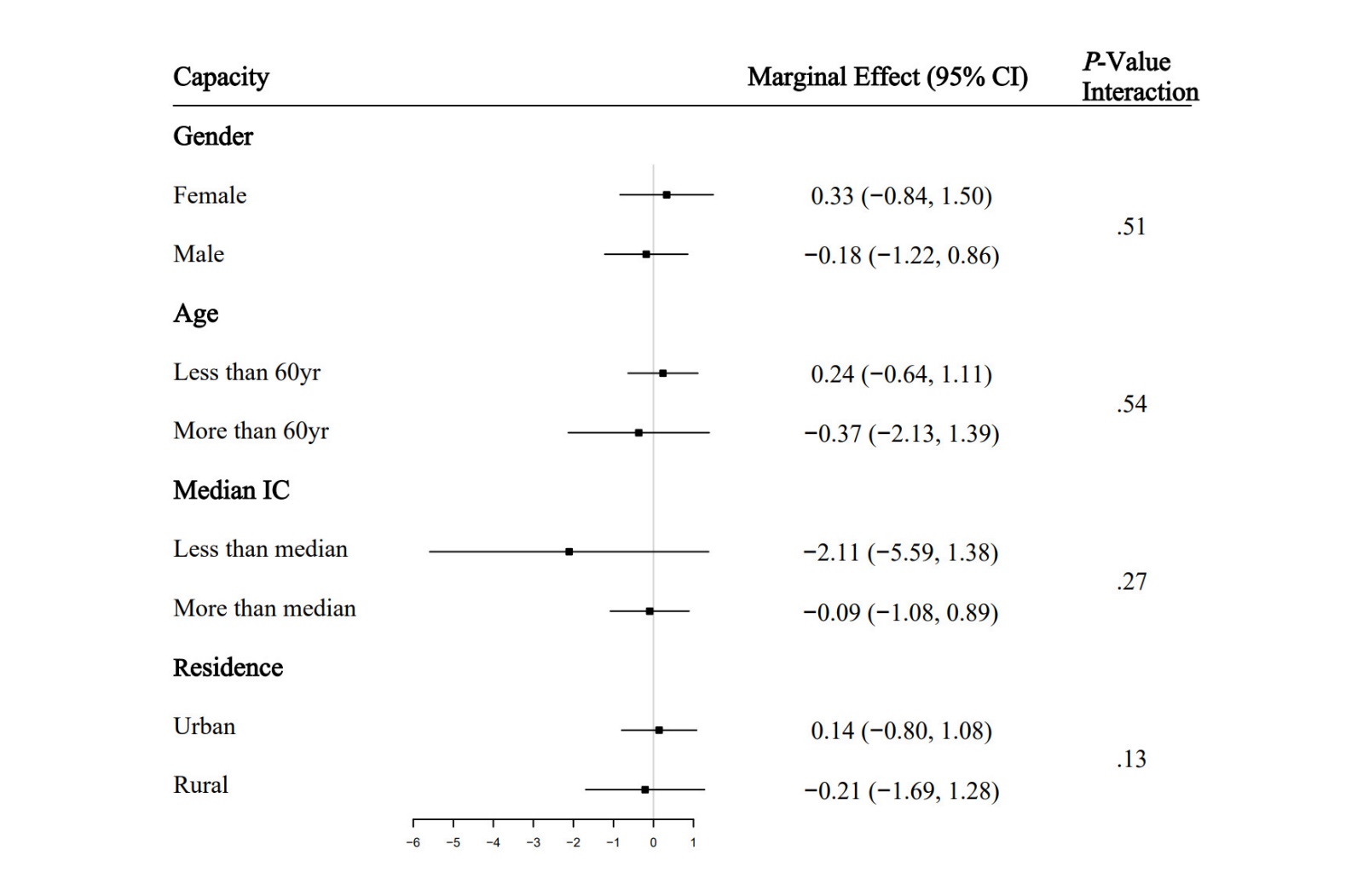


**Figure S11**. Subgroup analyses for the association between the intrinsic capacity with its psychological capacity and daily internet use by gender, age, residence, and median 2011 IC, adjusted for demographic variables (age, gender, residence, education level and annual household income) +lifestyle (drinking history, smoking history, social participation and MET-PA) + health status (BMI, hypertension, dyslipidemia, diabetes, cancer, chronic lung diseases, liver disease, heart diseases, stroke, kidney diseases, digestive diseases, psychiatric problems, memory-related diseases, arthritis or rheumatism, and asthma). The IC total score was adjusted using the residual method.

Abbreviations: IC, intrinsic capacity; CI, confidence intervals.


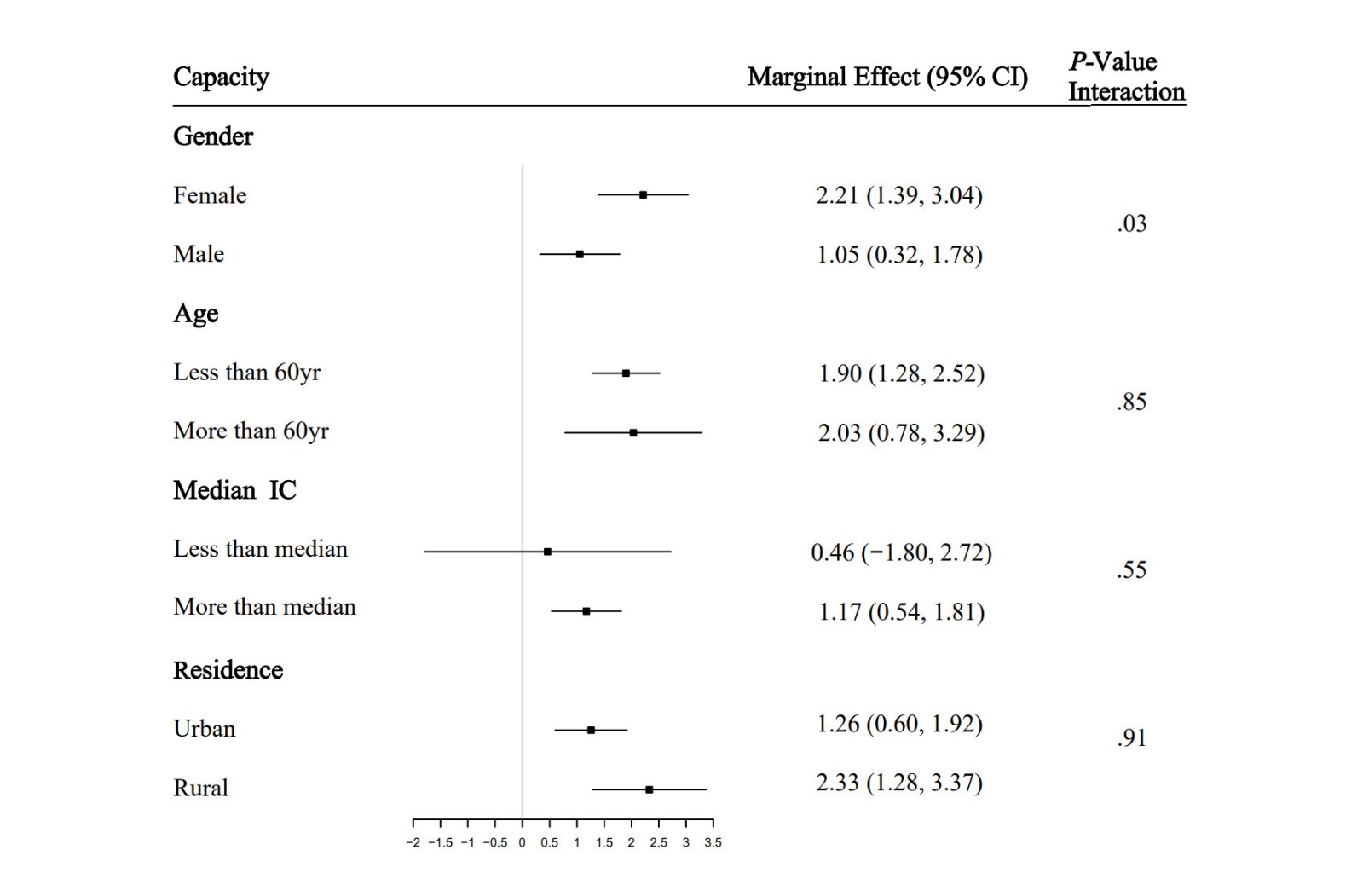


**Figure S12**. Subgroup analyses for the association between intrinsic capacity with cognitive capacity and daily internet use by gender, age, residence, and median 2011 IC, adjusted for demographic variables (age, gender, residence, education level and annual household income) +lifestyle (drinking history, smoking history, social participation and MET-PA) + health status (BMI, hypertension, dyslipidemia, diabetes, cancer, chronic lung diseases, liver disease, heart diseases, stroke, kidney diseases, digestive diseases, psychiatric problems, memory-related diseases, arthritis or rheumatism, and asthma). The IC total score was adjusted using the residual method.

Abbreviations: IC, intrinsic capacity; CI, confidence intervals.


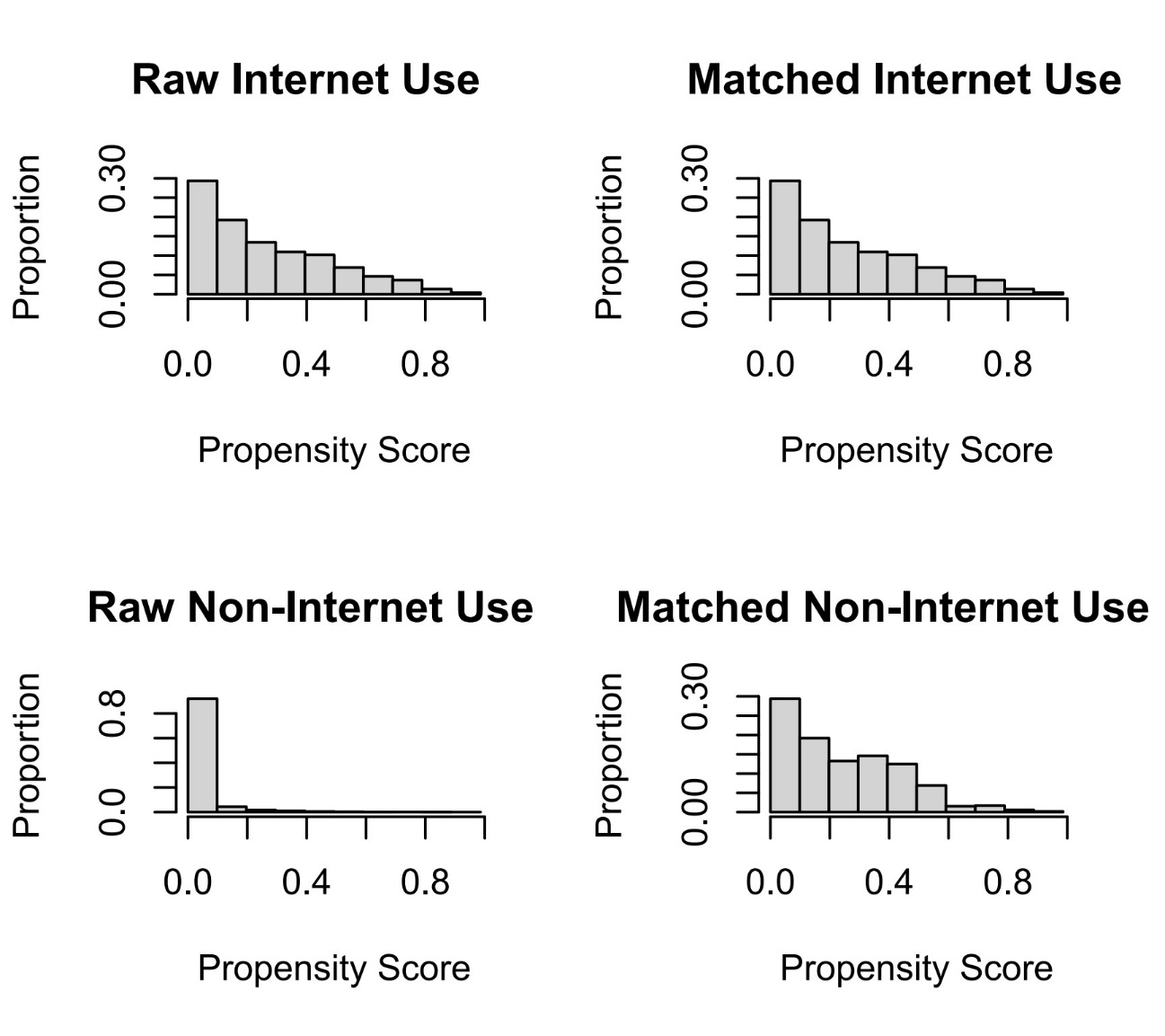


**Figure S13**. Propensity score matching analysis that compares the characteristics of two groups: internet users and non-users. Histograms illustrate the balancing of baseline characteristics achieved through the matching process, demonstrating that the distributions of propensity scores between the groups are equivalent and comparable, ensuring a valid comparison of the groups.
