# Supplementary material for: Association Between Daily Internet Use and Intrinsic Capacity Among Middle-Aged and Older Adults in China: Large Prospective Cohort Study
Source: J Med Internet Res. 2024 Nov 12;26:e54200. doi: 10.2196/54200 (PMC11599878; doi:10.2196/54200)
Supplement: Multimedia Appendix 2 [file jmir_v26i1e54200_app2.docx]

**Supplementary Methods**

**Intrinsic capacity data obtained from CHARLS**

**1. Walking speed time:** In CHARLS, each participant aged 60 and above was eligible for the timed walk test. In addition, prior to the actual test, participants were asked if they had any problems from recent surgery, injury, or other health conditions that might prevent them from walking. Only persons aged at least 60 years, willing to do the test, and able to walk (walking aids were permitted) were asked to walk 2.5 meters at their usual walking pace, twice. The time for both walks was recorded separately. In our analysis, we use the time (seconds) of the two trials.

**2. Chair-stand test:** The chair-stand test was used in CHARLS as a measure of physical performance, assessed the time required to rise from a chair to a full standing position five times with arms folded across the chest, with slower times reflecting worse function. The test incorporated the use of the respondent’s own armless, straight-backed chair. The time taken for the full stand was recorded in seconds. Participants were considered ineligible if they could not stand up without assistance; the use of walking aids, such as a walker or cane, was not permitted. The test was stopped if the person became too tired or short of breath, if the person used their hands, or if the nurse assessor felt concerned for the person’s safety.

**3. Balance:** Static balance was evaluated in CHARLS through three separate tests, which formed part of the Short Physical Performance Battery [1]. Participants were ineligible for the tests if they were chair-bound or wheelchair-based; if it became clear after discussion that they were too unsteady on their feet; if they found it painful to stand; or if either the nurse assessor or the participant, considered the test unsafe. We included data obtained for three components of the balance test: semi-tandem stand, full-tandem stand, and side-by-side stand: (a) Semi-tandem stand: Participants had to stand with the side of the heel of one foot touching the big toe of the other foot for at least 10 s. Participants unable to hold the position for 10 s scored one and no further tests were attempted. Those able to hold the position for 10 s moved on to the full-tandem stand; (b) Full-tandem stand: For this test, participants had to stand with the heel of one foot in front of and touching the toes of the other foot. Those aged 70 and above and able to hold the position for at least 30 seconds scored two points for this test; those aged below 70 and able to hold the position for at least 60 seconds scored two points for this test; those able to hold the position less than required time for certain age scored one point for this test; Those unable to hold this position scored no additional points; (c) Side-by-side stand: Participants were asked to stand with feet together, side-by-side, for at least 10 seconds, using their arms, bending their knees or moving their body to maintain balance, but not moving their feet. If the participant was unable to hold the position for 10 s, a score of zero was recorded and no further tests were attempted. Those able to hold the position for 10 seconds moved on to the semi-tandem stand. Note that semi-tandem stand and full-tandem stand are comparably more difficult than side-by-side stand, and in CHARLS 2011, a side-by-side stand was tested after the semi-tandem stand and full-tandem stand, with a low response rate. Therefore, we imputed side-by-side stand as one, if the participant was able to complete the semi-tandem stand and full-tandem stand; those unable to complete this test scored no additional points.

**4. Grip strength:** The grip strength test was used in CHARLS to test upper body strength. Handgrip strength (kg) of the dominant hand was assessed using a hand-held dynamometer. In the CHARLS 2011 wave, there were two measures for each hand. An average of the dominant hand was calculated for analysis (if both hands were reported as the dominant hand, we chose the larger measure). Any measurements carried out incorrectly or participants refused to perform the test were not included.

**5. Forced expiratory volume (FEV)**: Lung function was measured in CHARLS using a spirometer. Eligible participants were asked to stand or sit, take a deep breath, and blow into the spirometer as hard as they could. They were then required to repeat the procedure to give three technically satisfactory blows. The highest technically satisfactory measure of FEV was used in the analysis.

**6. Blood assay:** The CHARLS wave 2011 collected venous blood samples, and a complete blood count analysis was undertaken at local CDC laboratories (this included hemoglobin, hematocrit, white blood cell count, platelet counts, and mean corpuscular volume). Three tubes of venous blood were collected from each participant by medically trained staff from the China CDC, using a standard protocol. Detailed information on the technicalities of the blood analysis, the internal quality control, and the external quality assessment for the laboratory have been described on the website of CHARLS (http://charls.pku.edu.cn/index/en.html).We used hemoglobin in this study for validation analysis.

**7. Sensory:** Hearing and vision impairments were measured in CHARLS using self-report. Hearing status was assessed by asking participants to rate their hearing (using a hearing aid if they used one) as excellent, very good, good, fair, or poor. For vision, participants were also asked ‘How good is your eyesight for seeing things at a distance, like recognizing a friend across the street (with glasses or corrective lenses if you wear them)’ and ‘How good is your eyesight for seeing things up close, like reading ordinary newspaper print’. Response options (excellent/very good/good/fair–poor) were categorized as above.

**8. Cognition:** The CHARLS data include scores on two tests of cognitive function: episodic memory and intact mental status [2,3]. Memory recall data were based on the participant’s ability to recall the same list of words four minutes later (delayed recall). Episodic memory measures were obtained from delayed recall scores. Another cognitive measure was based on some components of the mental status questions of the Telephone Interview of Cognitive Status (TICS) battery [2], including the following: Serial 7 test, which requires the person to subtract 7 from 100 (up to five times); assessment of the person’s need of explanation, or use of an aid such as paper and pencil to undertake the serial 7 test; ability to accurately identify the current date (month, day and year) and season; and ability to redraw a picture shown to the person.

**9. Affect:** Affect was assessed using the ten-item Center for Epidemiological Studies-Depression (CES-D) scale [4, except for the sleep dimension which was considered independently (see the following measure). Six of the nine CES-D items (i.e., was bothered, felt hopeful, felt depressed, was happy, felt lonely, felt fearful) were considered to be depressed mood items, while the remaining three (i.e., everything was an effort, had trouble keeping mind, and could not get going) were regarded as somatic complaints items. In order to allow comparison with Beard et al.’ s [5] previous validation methodology, nine items collected in CHARLS referring to how the participant felt and behaved during the last week. Each item corresponded with four categorized answers, rarely or none of the time (< 1 day), some or a little of the time (1−2 days), occasionally or a moderate amount of the time (3−4 days), and most or all of the time (5−7 days). A summary CES-D score was derived by adding responses to all nine categorized questions.

**10. Sleep:** Three sleep items in CHARLS wave 2011 were included: sleep hours at night; nap minutes at noon; and sleep quality. Participants were asked about how many hours of actual sleep they got at night (average hours for one night, which may be shorter than the number of hours spent in bed) and how long they took a nap after lunch (minutes), on average, during the past month. For sleep quality, participants were asked to report the frequency of restless sleep during the last week with four categorized answers, rarely or none of the time (< 1 day), some or a little of the time (1 − 2 days), occasionally or a moderate amount of the time (3−4 days), and most or all of the time (5−7 days).

**References**

1. Guralnik JM, Simonsick EM, Ferrucci L, et al. A short physical performance battery assessing lower extremity function:

association with self-reported disability and prediction of mortality and nursing home admission. J Gerontol.

1994;49(2):M85-M94.

2. Lei X, Hu Y, McArdle JJ, Smith JP, Zhao Y. Gender differences in cognition among older adults in China. J Hum Resour.

2012;47(4):951-971.

3. Lei X, Smith JP, Sun X, Zhao Y. Gender differences in cognition in China and reasons for change over time: evidence from

CHARLS. J Econ Ageing. 2014;4:46-55.

4. Carleton RN, Thibodeau MA, Teale MJ, et al. The center for epidemiologic studies depression scale: a review with a

theoretical and empirical examination of item content and factor structure. PloS One. 2013;8(3):e58067.

5. Beard JR, Jotheeswaran AT, Cesari M, de Carvalho IA. The structure and predictive value of intrinsic capacity in a

longitudinal study of ageing. BMJ Open. 2019;9(11).
