# Supplementary material for: Association Between Daily Internet Use and Intrinsic Capacity Among Middle-Aged and Older Adults in China: Large Prospective Cohort Study
Source: J Med Internet Res. 2024 Nov 12;26:e54200. doi: 10.2196/54200 (PMC11599878; doi:10.2196/54200)
Supplement: Multimedia Appendix 3 [file jmir_v26i1e54200_app3.docx]

Supplemental Table S1-S5

| IC and five subdomains | Hypertension | |  | Dyslipidemia | |  | Diabetes or high blood sugar | |  | Cancer or malignant tumor | |  | Chronic lung diseases | |  | Liver disease | |  | Heart attack | |
| --- | --- | --- | --- | --- | --- | --- | --- | --- | --- | --- | --- | --- | --- | --- | --- | --- | --- | --- | --- | --- |
|  | Rho | *P* Value |  | Rho | *P* Value |  | Rho | *P* Value |  | Rho | *P* Value |  | Rho | *P* Value |  | Rho | *P* Value |  | Rho | *P* Value |
|  |  |  |  |  |  |  |  |  |  |  |  |  |  |  |  |  |  |  |  |  |
|  |  |  |  |  |  |  |  |  |  |  |  |  |  |  |  |  |  |  |  |  |
| IC total score | -0.075 | <.001 |  | 0.017 | .06 |  | -0.024 | .01 |  | -0.032 | <.001 |  | -0.118 | <.001 |  | -0.028 | .002 |  | -0.101 | <.001 |
| Locomotion | -0.117 | <.001 |  | -0.035 | <.001 |  | -0.047 | <.001 |  | -0.008 | .35 |  | -0.045 | <.001 |  | -0.009 | .30 |  | -0.105 | <.001 |
| Sensory | -0.080 | <.001 |  | -0.035 | <.001 |  | -0.054 | <.001 |  | -0.024 | .01 |  | -0.093 | <.001 |  | -0.052 | <.001 |  | -0.100 | <.001 |
| Vitality | -0.049 | <.001 |  | 0.021 | .02 |  | -0.027 | .003 |  | -0.030 | .001 |  | -0.072 | <.001 |  | 0.008 | .37 |  | -0.076 | <.001 |
| Psychological | -0.055 | <.001 |  | -0.034 | <.001 |  | -0.039 | <.001 |  | -0.032 | <.001 |  | -0.101 | <.001 |  | -0.059 | <.001 |  | -0.107 | <.001 |
| Cognitive | -0.018 | .04 |  | 0.063 | <.001 |  | 0.027 | .002 |  | -0.009 | .29 |  | -0.050 | <.001 |  | 0.002 | .79 |  | -0.003 | .76 |

Table S1. Spearman correlation matrix calculated the correlation between 14 chronic diseases and IC score in 2011.

| IC and five subdomains | Stroke | |  | Kidney disease | |  | Stomach or other digestive disease | |  | Emotional or psychiatric problems | |  | Memory-related disease | |  | Arthritis or rheumatism | |  | Asthma | |
| --- | --- | --- | --- | --- | --- | --- | --- | --- | --- | --- | --- | --- | --- | --- | --- | --- | --- | --- | --- | --- |
|  | Rho | *P* Value |  | Rho | *P* Value |  | Rho | *P* Value |  | Rho | *P* Value |  | Rho | *P* Value |  | Rho | *P* Value |  | Rho | *P* Value |
|  |  |  |  |  |  |  |  |  |  |  |  |  |  |  |  |  |  |  |  |  |
|  |  |  |  |  |  |  |  |  |  |  |  |  |  |  |  |  |  |  |  |  |
| IC total score | -0.063 | <.001 |  | -0.066 | <.001 |  | -0.133 | <.001 |  | -0.064 | <.001 |  | -0.055 | <.001 |  | -0.230 | <.001 |  | -0.093 | <.001 |
| Locomotion | -0.084 | <.001 |  | -0.023 | .01 |  | -0.030 | .001 |  | -0.036 | <.001 |  | -0.060 | <.001 |  | -0.096 | <.001 |  | -0.050 | <.001 |
| Sensory | -0.053 | <.001 |  | -0.068 | <.001 |  | -0.120 | <.001 |  | -0.046 | <.001 |  | -0.056 | <.001 |  | -0.179 | <.001 |  | -0.076 | <.001 |
| Vitality | -0.035 | <.001 |  | -0.009 | 0.33 |  | -0.063 | <.001 |  | -0.031 | <.001 |  | -0.018 | .043 |  | -0.127 | <.001 |  | -0.057 | <.001 |
| Psychological | -0.044 | <.001 |  | -0.090 | <.001 |  | -0.169 | <.001 |  | -0.070 | <.001 |  | -0.032 | <.001 |  | -0.206 | <.001 |  | -0.072 | <.001 |
| Cognitive | -0.016 | .07 |  | -0.018 | .04 |  | -0.066 | <.001 |  | -0.036 | <.001 |  | -0.029 | .001 |  | -0.140 | <.001 |  | -0.041 | <.001 |

Rho: Spearman's rank correlation coefficient (Statistical dependence between the rankings of two variables).

IC: intrinsic capacity

| IC and five subdomains | Hypertension | |  | Dyslipidemia | |  | Diabetes or high blood sugar | |  | Cancer or malignant tumor | |  | Chronic lung diseases | |  | Liver disease | |  | Heart attack | |
| --- | --- | --- | --- | --- | --- | --- | --- | --- | --- | --- | --- | --- | --- | --- | --- | --- | --- | --- | --- | --- |
|  | Rho | *P* Value |  | Rho | *P* Value |  | Rho | *P* Value |  | Rho | *P* Value |  | Rho | *P* Value |  | Rho | *P* Value |  | Rho | *P* Value |
|  |  |  |  |  |  |  |  |  |  |  |  |  |  |  |  |  |  |  |  |  |
|  |  |  |  |  |  |  |  |  |  |  |  |  |  |  |  |  |  |  |  |  |
| IC total score | 0.0002 | .98 |  | 0.023 | .01 |  | -0.005 | .56 |  | -0.004 | .67 |  | 0.022 | .01 |  | 0.028 | .001 |  | 0.031 | <.001 |
| Locomotion | -0.009 | .31 |  | 0.002 | .82 |  | -0.010 | .27 |  | -0.016 | .07 |  | -0.023 | .01 |  | 0.003 | .73 |  | 0.003 | .78 |
| Sensory | 0.018 | .04 |  | 0.014 | .12 |  | 0.008 | .34 |  | -0.001 | .92 |  | 0.036 | <.001 |  | 0.025 | .01 |  | 0.028 | .001 |
| Vitality | -0.015 | .09 |  | -0.006 | .51 |  | 0.014 | .12 |  | 0.010 | .26 |  | -0.019 | .04 |  | 0.015 | .09 |  | -0.010 | .25 |
| Psychological | 0.014 | .11 |  | 0.018 | .05 |  | 0.001 | .91 |  | -0.001 | .88 |  | 0.027 | .002 |  | 0.014 | .11 |  | 0.019 | .03 |
| Cognitive | -0.006 | .50 |  | 0.017 | .06 |  | -0.019 | .04 |  | -0.011 | .23 |  | 0.012 | .17 |  | 0.021 | .02 |  | 0.010 | .24 |

| IC and five subdomains | Stroke | |  | Kidney disease | |  | Stomach or other digestive disease | |  | Emotional or psychiatric problems | |  | Memory-related disease | |  | Arthritis or rheumatism | |  | Asthma | |
| --- | --- | --- | --- | --- | --- | --- | --- | --- | --- | --- | --- | --- | --- | --- | --- | --- | --- | --- | --- | --- |
|  | Rho | *P* Value |  | Rho | *P* Value |  | Rho | *P* Value |  | Rho | *P* Value |  | Rho | *P* Value |  | Rho | *P* Value |  | Rho | *P* Value |
|  |  |  |  |  |  |  |  |  |  |  |  |  |  |  |  |  |  |  |  |  |
|  |  |  |  |  |  |  |  |  |  |  |  |  |  |  |  |  |  |  |  |  |
| IC total score | -0.003 | .71 |  | 0.032 | <.001 |  | 0.047 | <.001 |  | 0.028 | <.001 |  | 0.003 | .72 |  | 0.052 | <.001 |  | 0.022 | .01 |
| Locomotion | -0.008 | .39 |  | -0.002 | .78 |  | 0.008 | .36 |  | -0.00003 | .97 |  | 0.000 | >.99 |  | -0.009 | .33 |  | 0.003 | .75 |
| Sensory | 0.009 | .29 |  | 0.014 | .10 |  | 0.028 | .002 |  | 0.010 | .25 |  | 0.029 | .001 |  | 0.027 | .002 |  | 0.029 | .001 |
| Vitality | 0.001 | .90 |  | 0.004 | .62 |  | 0.004 | .64 |  | 0.004 | .64 |  | -0.011 | .23 |  | 0.007 | .43 |  | -0.024 | .01 |
| Psychological | 0.008 | .36 |  | 0.011 | .23 |  | 0.037 | <.001 |  | 0.019 | .03 |  | -0.001 | .91 |  | 0.042 | <.001 |  | 0.031 | <.001 |
| Cognitive | -0.002 | .80 |  | 0.024 | .01 |  | 0.024 | .01 |  | 0.021 | .02 |  | -0.004 | .64 |  | 0.033 | <.001 |  | 0.014 | .11 |

Table S2. Spearman correlation matrix calculated the correlation between 14 chronic diseases and IC score in 2015.

Rho: Spearman's rank correlation coefficient (Statistical dependence between the rankings of two variables).

IC: intrinsic capacity

Table S3. IC and five subdomains sensitivity analysis Model 1-4.

| IC and five subdomains | Model 1 | |  | Model 2 | |  | Model 3 | |  | Model 4 | |
| --- | --- | --- | --- | --- | --- | --- | --- | --- | --- | --- | --- |
|  | Marginal effect  (95%CI) | *P* value |  | Marginal effect  (95%CI) | *P* value |  | Marginal effect (95%CI) | *P* value |  | Marginal effect (95%CI) | *P* value |
| IC total score | 1.58  (1.04,2.11) | <.001 |  | 1.50  (0.92,2.09) | <.001 |  | 1.58  (0.69,2.47) | <.001 |  | 1.90  (1.12,2.67) | <.001 |
| Locomotion | 1.30  (0.59,2.02) | <.001 |  | 1.18  (0.13,2.23) | .03 |  | 1.31  (0.12,2.51) | .03 |  | 1.47  (0.44,2.50) | .01 |
| Sensory | 1.32  (0.62,2.03) | <.001 |  | 1.87  (0.43,3.30) | .01 |  | 1.12  (-0.06,2.29) | .06 |  | 1.72  (0.70,2.73) | <.001 |
| Vitality | 1.17  (0.66,1.68) | <.001 |  | 3.07  (1.94,4.19) | <.001 |  | 1.11  (0.27,1.96) | .01 |  | 1.17  (0.44,1.90) | <.001 |
| Psychological | 0.04  (-0.76,0.84) | .92 |  | -1.62  (-2.96, -0.29) | .02 |  | 0.05  (-1.29,1.38) | .94 |  | 0.52  (-0.63,1.67) | .38 |
| Cognitive | 1.56  (1.00,2.12) | <.001 |  | -0.15  (-1.10,0.79) | .75 |  | 1.81  (0.87,2.75) | <.001 |  | 1.57  (0.75,2.38) | <.001 |

Model 1 was used to evaluate the original IC total score of the middle-aged and older population using the Internet in 2011.

Model 2 was used to evaluate the IC total score of the middle-aged and older population using the Internet after adjusting for changes (increase or decrease) in IC total scores in 2015 compared to 2011 using the Mills ratio.

Model 3 was used to evaluate the IC total score of the middle-aged and older population (constant users) who used the Internet in 2011 and 2013.

Model 4 was used to evaluate the IC total score of the middle-aged and older population (new users) who did not use the Internet in 2011 but used it in 2013.

Multivariate linear regression analysis adjusted demographic variables (age, gender, residence, education level, and annual household income) +lifestyle (drinking history, smoking history, social participation and MET-PA) +health status (BMI, hypertension, dyslipidemia, diabetes, cancer, chronic lung diseases, liver disease, heart diseases, stroke, kidney diseases, digestive diseases, psychiatric problems, memory-related diseases, arthritis or rheumatism, and asthma). The IC total score was adjusted using the residual method.

IC: intrinsic capacity; CI: confidence interval.

Table S4. IC and five subdomains sensitivity analysis Model 5-8.

| IC and five subdomains | Model 5 | |  | Model 6 | |  | Model 7 | |  | Model 8 | |
| --- | --- | --- | --- | --- | --- | --- | --- | --- | --- | --- | --- |
|  | Marginal effect  (95%CI) | *P* value |  | Marginal effect  (95%CI) | *P* value |  | Marginal effect (95%CI) | *P* value |  | Marginal effect (95%CI) | *P* value |
| IC total score | 1.55  (1.02,2.08) | <.001 |  | 1.59  (1.06,2.13) | <.001 |  | 1.72  (1.19,2.25) | <.001 |  | 1.66  (0.80,1.95) | <.001 |
| Locomotion | 1.30  (0.59,2.01) | <.001 |  | 1.37  (0.66,2.08) | <.001 |  | 1.30  (0.59,2.00) | <.001 |  | 1.02  (0.65,2.15) | .02 |
| Sensory | 1.33  (0.62,2.03) | <.001 |  | 1.36  (0.65,2.07) | <.001 |  | 1.13  (0.43,1.84) | .002 |  | 1.06  (-0.31,1.60) | .07 |
| Vitality | 1.01  (0.56,1.46) | <.001 |  | 1.19  (0.68,1.69) | <.001 |  | 1.22  (0.72,1.73) | <.001 |  | 1.16  (-0.45,1.56) | .13 |
| Psychological | 0.05  (-0.75,0.85) | .91 |  | 0.08  (-0.72,0.88) | .84 |  | 0.06  (-0.74,0.86) | .88 |  | 0.23  (-0.84, 0.96) | .69 |
| Cognitive | 1.56  (0.99,2.12) | <.001 |  | 1.55  (0.99,2.12) | <.001 |  | 1.98  (1.42,2.55) | <.001 |  | 1.38  (0.83,1.92) | <.001 |

Model 5 was used to evaluate the IC total score of the middle-aged and older population who use the Internet after excluding the extreme IC total score.

Model 6 was used to evaluate the total IC score of the middle-aged and older population who use the Internet after excluding the population with memory related diseases (Alzheimer's disease, brain atrophy, Parkinson's disease) in 2011.

Model 7 was used to evaluate the IC total score of the middle-aged and older population who use the Internet in 2015 as the baseline year.

Model 8 was used to evaluate the IC total score of the middle-aged and older population who use the Internet after the propensity score matching equilibrium.

Multivariate linear regression analysis adjusted demographic variables (age, gender, residence, education level, and annual household income) +lifestyle (drinking history, smoking history, social participation and MET-PA) +health status (BMI, hypertension, dyslipidemia, diabetes, cancer, chronic lung diseases, liver disease, heart diseases, stroke, kidney diseases, digestive diseases, psychiatric problems, memory-related diseases, arthritis or rheumatism, and asthma). The IC total score was adjusted using the residual method.

IC: intrinsic capacity; CI: confidence interval.

Table S5. The Mediation analysis of 14 chronic diseases, social participation, and MET-PA with IC.

| All-cause | IC total score | |  | Locomotion | |  | Sensory | |  | Vitality | |  | Psychological | |  | Cognitive | |
| --- | --- | --- | --- | --- | --- | --- | --- | --- | --- | --- | --- | --- | --- | --- | --- | --- | --- |
|  | Mediation proportion (%) | *P* Value |  | Mediation proportion (%) | *P* Value |  | Mediation proportion (%) | *P* Value |  | Mediation proportion (%) | *P* Value |  | Mediation proportion (%) | *P* Value |  | Mediation proportion (%) | *P* Value |
|  |  |  |  |  |  |  |  |  |  |  |  |  |  |  |  |  |  |
|  |  |  |  |  |  |  |  |  |  |  |  |  |  |  |  |  |  |
| Hypertension | -0.50  (-1.87,0.56) | .32 |  | 0.04  (-0.38,0.76) | .72 |  | -0.60  (-2.80,0.92) | .39 |  | -0.15  (-1.00,0.29) | .44 |  | -0.95  (-32.71,27.28) | .71 |  | -0.3  (-1.45,0.44) | .39 |
| Dyslipidemia | -0.17  (-1.96,1.51) | .82 |  | -0.01  (-0.96,0.86) | .94 |  | -0.26  (-3.90,3.00) | .85 |  | -0.01  (-0.64,0.50) | .93 |  | -0.26  (-58.59,57.39) | .96 |  | -0.01  (-0.74,0.67) | .94 |
| Diabetes | -0.22  (-1.43,0.64) | .54 |  | -0.16  (-1.81, 1.00) | .67 |  | -0.17  (-1.75,0.81) | .62 |  | -0.01  (-0.61,0.39) | .86 |  | -1.21  (-47.44,43.98) | .78 |  | -0.02  (-0.70,0.41) | .81 |
| Cancer | 0.002  (-0.30,0.32) | .95 |  | 0.008  (-0.59,0.66) | .92 |  | 0.05  (-0.79,1.15) | .82 |  | -0.05  (-0.80,0.58) | .78 |  | 0.06  (-13.73,13.67) | .92 |  | -0.003  (-0.44,0.35) | .94 |
| Chronic lung diseases | 0.09  (-0.43,0.81) | .63 |  | 0.21  (-0.35,1.31) | .40 |  | 0.38  (-0.55,1.99) | .37 |  | 0.02  (-0.72,1.03) | .84 |  | 0.01  (-9.51,10.54) | .84 |  | 0.05  (-0.26,0.66) | .63 |
| Liver diseases | -0.10  (-1.20,0.75) | .73 |  | 0.30  (-0.69,2.00) | .48 |  | -1.39  (1.99,0.20) | .09 |  | 0.23  (-0.54,1.63) | .48 |  | -3.31  (-84.75,78.76) | .51 |  | 0.17  (-0.52,1.25) | .54 |
| Heart diseases | -0.004  (-0.68,0.65) | .98 |  | 0.01  (-0.97,1.01) | .98 |  | 0.001  (-0.85,0.89) | >.99 |  | 0.001  (-0.42,0.53) | .97 |  | 0.0001  (-13.34,10.48) | >.99 |  | 0.001  (-0.39,0.41) | .97 |
| Stroke | 0.35  (-0.37,1.35) | .32 |  | 0.91  (-0.20,2.91) | .11 |  | 0.25  (-0.23,1.36) | .29 |  | 0.55  (-0.12,1.72) | .12 |  | 0.73  (-20.17,19.67) | .60 |  | 0.2  (-0.13,0.86) | .25 |
| Kidney diseases | 0.33  (-0.07,1.13) | .13 |  | -0.02  (-0.79,0.65) | .92 |  | 0.92  (0.04,2.93) | .04 |  | 0.56  (0.02, 1.68) | .04 |  | 2.91  (-53.84,71.48) | .49 |  | -0.18  (-0.91,0.32) | .42 |
| Digestive diseases | -0.04  (-1.06,0.94) | .94 |  | 0.0003  (-0.45,0.46) | >.99 |  | 0.05  (-1.46,1.69) | .93 |  | 0.02  (-0.52,0.70) | .86 |  | 0.05  (-37.55,32.80) | .99 |  | 0.01  (-0.65,0.67) | .96 |
| Psychiatric problems | 0.33  (0.03,0.85) | .02 |  | 0.30  (-0.17,1.07) | .21 |  | 0.14  (-0.33,0.84) | .49 |  | 0.05  (-0.33,0.53) | .73 |  | 1.39  (-29.48,33.92) | .52 |  | 0.41  (0.06, 1.00) | .01 |
| Memory-related diseases | -0.20  (-1.27,0.68) | .60 |  | -0.01  (-0.99,0.87) | .95 |  | -0.06  (-1.46,1.21) | .91 |  | -0.01  (-0.44,0.37) | .92 |  | -0.02  (-15.24,12.83) | .98 |  | -0.04  (-0.80,0.62) | .88 |
| Arthritis or rheumatism | 1.42  (0.30,3.02) | .01 |  | -0.36  (-1.58,0.37) | .30 |  | 1.48  (0.31,4.05) | .01 |  | 0.48  (-0.53,2.04) | .30 |  | 6.03  (-101.07,108.75) | .47 |  | 0.99  (0.21, 2.36) | .01 |
| Asthma | 0.05  (-0.32,0.54) | .69 |  | -0.0001  (-0.56,0.50) | >.99 |  | 0.17  (-0.23,1.08) | .37 |  | 0.49  (-0.53,2.04) | .39 |  | 0.01  (-6.08,6.99) | .97 |  | 0.03  (-0.21,0.46) | .67 |
| Social participation | 28.78  (21.24,40.33) | <.001 |  | 26.98  (16.96,44.56) | <.001 |  | 34.41  (20.60,71.73) | <.001 |  | 13.15  (6.16, 24.34) | <.001 |  | 39.45  (-710.65,781.57) | .47 |  | 23.44  (16.51,33.17) | <.001 |
| Vigorous activities | -1.05  (-3.86,0.21) | .12 |  | -5.75  (-14.48, -0.68) | .03 |  | -0.25  (-12.08,9.12) | .82 |  | -1.44  (-6.12,0.18) | .08 |  | -1.78  (-49.20,44.87) | .65 |  | 0.33  (-0.80,2.15) | .49 |
| Moderate activities | 2.76  (-3.11,9.88) | .32 |  | 4.99  (-2.16,13.27) | .16 |  | 3.94  (-21.69,38.63) | .21 |  | 1.09  (-0.73,5.17) | .22 |  | 0.08  (-20.60,22.35) | .93 |  | 2.3  (-1.62,7.66) | .22 |
| Leisure  activities | 4.26  (1.33,9.79) | .002 |  | 4.74  (1.74,10.34) | <.001 |  | 2.07  (-15.44,23.58) | .40 |  | 3.82  (1.07, 11.73) | .01 |  | -4.38  (-103.59,111.87) | .58 |  | 2.83  (0.78, 6.35) | .002 |

The total effect was determined by the structural equation model (SEMs). The mediation proportion was calculated based on the association between daily internet use and IC (linear model). All models were conducted by adjusting for demographic variables (age, gender, residence, education level, and annual household income) +lifestyle (drinking history, smoking history, social participation, and MET-PA) +health status (BMI, hypertension, dyslipidemia, diabetes, cancer, chronic lung diseases, liver disease, heart diseases, stroke, kidney diseases, digestive diseases, psychiatric problems, memory-related diseases, arthritis or rheumatism, and asthma). The IC total score was adjusted using the residual method.

IC: intrinsic capacity; CI: confidence interval.
